# Species Choice and Model Use: Reviving Research on Human Development

**DOI:** 10.1007/s10739-024-09775-7

**Published:** 2024-07-29

**Authors:** Nick Hopwood

**Affiliations:** Department of History and Philosophy of Science, University of Cambridge, Free School Lane, Cambridge, CB2 3RH UK

**Keywords:** Biobanks, Digital anatomy, Human developmental biology, Model organisms, Postgenomic science, Stem-cell-based embryo models

## Abstract

While model organisms have had many historians, this article places studies of humans, and particularly our development, in the politics of species choice. Human embryos, investigated directly rather than via animal surrogates, have gone through cycles of attention and neglect. In the past 60 years they moved from the sidelines to center stage. Research was resuscitated in anatomy, launched in reproductive biomedicine, molecular genetics, and stem-cell science, and made attractive in developmental biology. I explain this surge of interest in terms of rivalry with models and reliance on them. The greater involvement of medicine in human reproduction, especially through in vitro fertilization, gave access to fresh sources of material that fed critiques of extrapolation from mice and met demands for clinical relevance or “translation.” Yet much of the revival depended on models. Supply infrastructures and digital standards, including biobanks and virtual atlases, emulated community resources for model organisms. Novel culture, imaging, molecular, and postgenomic methods were perfected on less precious samples. Toing and froing from the mouse affirmed the necessity of the exemplary mammal and its insufficiency justified inquiries into humans. Another kind of model—organoids and embryo-like structures derived from stem cells—enabled experiments that encouraged the organization of a new field, human developmental biology. Research on humans has competed with and counted on models.

Physicians and scientists have long been curious about human development, but our tiny embryos are usually hidden in precious bodies. Researchers have either made do with the little human material they could collect or studied accessible species as surrogates. The former strategy has passed into and out of vogue. In the early 19th century leading anatomists and physiologists reckoned the few human preparations so poor that chicks and domestic mammals taught more about us. In the early 20th century the first research institute dedicated to embryology, a department of the Carnegie Institution of Washington, was founded to focus on humans. Fifty years later studies of frogs, flies, and mice took over (Hopwood 2009, 2015, 2019). Today human developmental biology is booming as a path to alleviating infertility, preventing congenital disease, and knowing ourselves. What has changed through this latest cycle in the politics of species choice (Gilbert 2009; Hopwood 2011)?

Such questions have driven many histories of the rise and fall of experimental and model organisms (Mitman and Fausto-Sterling 1992; Kohler 1994; de Chadarevian 1998; Gurdon and Hopwood 2000; Rader 2004; Leonelli 2007; Ankeny and Leonelli 2020; Dietrich et al. 2020; Reiß 2020; Bolman 2022). They have hardly been addressed for research on human beings. Accounts of the postwar institutionalization of human genetics, for example, tend to assume an interest in our own species rather than thematize competition with investigations of other organisms (but see de Chadarevian 2020, chap. 1). That assumption reflects attitudes in hospitals and public health. But in the many fields that straddle medicine and academic biology, veterinary medicine, or agricultural science, the power of patrons and access to resources have determined the balance. Studies in humans were facilitated by medical relevance and public curiosity and stymied by inaccessibility and controversy (Panofsky 2014; Nelson 2018).

Among the sciences that provided niches for embryology some have defaulted to humans, especially anatomy with help from gynecology. The recent revival of research on human development marks more of a departure for disciplines that once concentrated on model organisms, such as reproductive physiology, molecular genetics, and developmental biology. So histories are split. Anatomists trace predecessors to a major work of the 1880s or the foundation of the Carnegie Department in 1914; some ascribe the “rebirth of human embryology” in the early 21st century to anatomy alone. By contrast, reproductive and developmental biologists, stem-cell researchers, and the social scientists who study them begin with human in vitro fertilization (IVF) in the postwar era, but are apt to disregard anatomy as merely descriptive.^1^ A gulf has also separated work on the conceptus in the first week after fertilization, using donations from fertility clinics, from work on embryonic stages after implantation in the uterus, employing material from terminations of pregnancy.^2^ Only a broad view across networks, processes, and agendas can recognize the multiple sources of change, identify common features, and assess the extent to which enterprises are coming together. This perspective will allow analysis of the range of approaches to species choice and model use.^3^

On the one hand, I shall suggest, the latest upswing in studies of human development was species-specific: it was driven by medical supply and demand. Political lobbying and government regulation made preimplantation stages from IVF available as never before, despite antiabortionism. Engagement with clinicians and patients also secured supplies of postimplantation embryos. (Telling this story will involve mention of miscarriage and other potentially distressing complications of reproduction.) The market for results expanded as funders insisted on clinical payoffs or “translation.” Molecular and postgenomic methods eased shifts between species and let smaller samples be tested. Investigators dared to suggest that they would discover what makes us human.^4^

On the other hand, I shall argue, models have not only been studied as surrogate humans; they have also enabled research directly on human embryos, and never more than today. Human embryologists had always applied knowledge and skills from other amniotes, notably chicks, rabbits, dogs, pigs, and monkeys, but the mouse became a constant reference point. By the late 20th century mice served as a test bed for techniques, a source of knowledge, and an exemplar of the community resources that suit a species to digital, data-centric, and imaging science. Yet to warrant studying humans, many scientists and funders had to be persuaded against simply extrapolating from mice. Researchers did not reject the model, but emphasized its imperfection. They constantly rediscovered adequacy (confirmed by concordant results) and insufficiency (demonstrated by divergent ones). They supplemented the dominant model with additional mammals.^5^

New surrogates—embryo models derived from stem cells—are human but not (yet) embryos, and so have complementary strengths and weaknesses. Subject to ongoing ethical negotiations, they for the first time permit systematic experimentation on structures that resemble human embryos or their parts. They are a constant reminder of what cannot be done in humans directly, and they hold out the prospect of independent work. Developmental biologists have begun to claim that, in addressing some fundamental biological questions, human in vitro systems are the best models.^6^

The article sets the scene with a sketch of the rise of human embryological anatomy to its mid-20th-century heyday and subsequent marginalization by developmental biology. It reconstructs a history of in vitro culture, with which the renaissance of research on human embryos began around 1970, and then also of projects from around 1990 to digitize old collections, use biobanked postimplantation material to survey gene expression, manipulate cultured embryos, and establish stem-cell models. It concentrates on the United States and the United Kingdom, where researchers and institutions have played the largest international roles, but encompasses an ever more globalized field with significant interventions from continental Europe, Japan, and Israel. Throughout I highlight the changing practices and arguments that promoted research on humans and relate these to model use. In this way I explain the articulation of a novel disciplinary identity, human developmental biology.^7^

## Rise and Fall

Minute and inaccessible, human embryos are among the hardest to study. They long had a secure place in anatomy, which prized early stages, and in midwifery, where interest grew as birth neared. But constructing a developmental series was challenging when rare objects mostly came from pregnancy losses, their ages unknown and normality in doubt (Hopwood 2000). Influential anatomists and physiologists disparaged the “isolated evidence of mostly malformed early human eggs” (Wagner 1839, p. 91). Fortunately, or so it seemed, the first half of the 19th century taught the similarity of development across the vertebrates. Researchers bet that work on domestic mammals and the chick would best “elucidate the early genesis of the human embryo” (Wagner 1839, p. 91; Hopwood 2015, chaps. 2–3).

From 1860 Darwinists politicized claims to similarity as they mobilized vertebrate embryos to argue for human evolution (Hopwood 2015, chaps. 5–8). Provoked to stress the disparities, anatomists set up a subfield to study our embryos for their own sake. Wilhelm His applied serial sectioning and wax reconstruction, which he had invented to access internal structure in the chick, to preparations donated by physicians. Published through the 1880s, his books and models covered the third to the eighth week of development and inspired the accumulation of a vast amount of data (Hopwood 1999, 2000, 2002, 2015, chaps. 6–8, 10).

On the eve of World War I a German-American handbook of human embryology ran to some 1,500 pages. It went, like textbooks, from the germ cells to organogenesis, plus one chapter each on the placenta and pathology. Unlike them, it left gaps rather than draw from surrogates. The introduction accepted that, “[c]onsidered purely objectively and scientifically the embryology of man has no more interest than that of any other […] vertebrate,” but rehearsed the traditional justifications: “the fact that we are human beings” and “importance to physicians” (Keibel and Mall 1910–12, 1: xviii). Anatomists still resorted to accessible species and among mammals prominently the pig (e.g., Patten 1927; Heuser and Streeter 1929).

Around the same time an experimental orientation, chiefly using amphibians and sea urchins, began to monopolize embryological attention (Hopwood 2009). But in 1914 the Carnegie Institution of Washington, a major private funder of science before the federal government was much involved, recognized the human research with a Department of Embryology at Johns Hopkins University in Baltimore. The founding director, anatomist Franklin Mall, lured the Carnegie president with optimism about deploying his mentor His’s techniques to enlarge understanding and shed light on miscarriage, “sterility,” and other pathologies. Above all Mall promised a rational approach to the body: “Gross human anatomy […] is bankrupt,” he wrote. “It is made solvent through embryology” (Mall 1911, p. 348; 1913; Noe 2004; Morgan 2009).

**Fig. 1:**
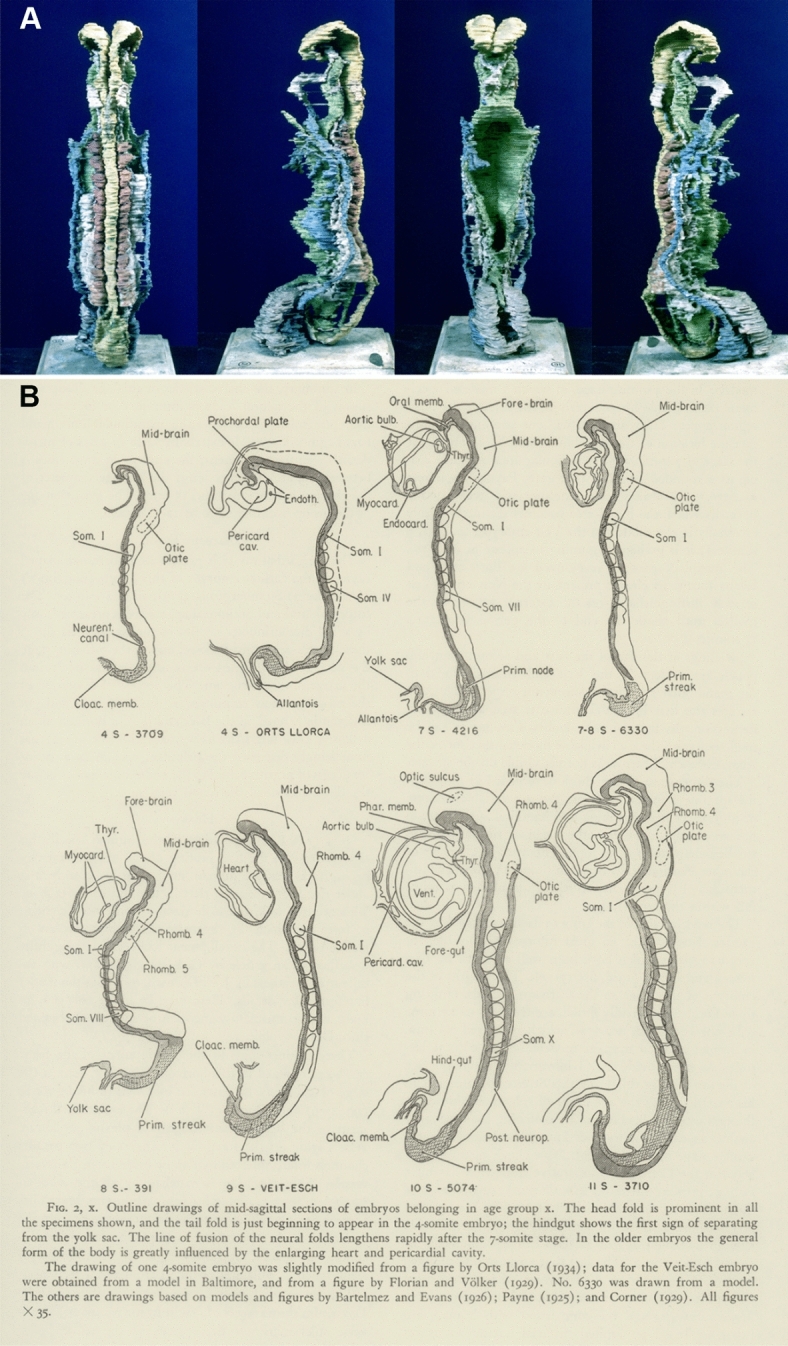
Embryos of horizon X (Carnegie Stage 10), 4 to 12 somites. **A** A plaster reconstruction of an 11-somite embryo (Carnegie no. 3710) by Osborne Heard, seen in four views. Human Developmental Anatomy Center, National Museum of Health and Medicine, Silver Spring, MD. **B** Outline drawings of mid-sagittal sections of eight embryos (with somite and collection numbers or names) in *Contributions to Embryology*. They show how much changed through the age group. The last is 3710, which originated in a spontaneous abortion and was donated by a Dr. E. Rice to the University of Chicago. From Heuser and Corner (1957, p. 33). Carnegie Science

The department extended and refined the developmental series. Mall and his successor George Streeter amassed via medical-school alumni and clinical colleagues 10,000 specimens from patients undergoing miscarriages, terminations, and other operations, and from postmortems. Hundreds were serially sectioned and reconstructed in plaster (Fig. 1A). The house journal published 38 lavish volumes mostly on humans. A few explored “racial embryology” (Morgan 2009, pp. 168–185).

When Mall began, the two weeks following fertilization had never been observed; the timing of ovulation was uncertain and the menstrual cycle still poorly understood. Then Hopkins scientists turned rhesus macaques into laboratory organisms and a yardstick for humans. This monkey gynecology provided direct evidence for mid-cycle ovulation, and let early cleavage in culture be filmed (Wilson 2012). When Streeter set up “horizons,” or age groups representing levels of structural organization (later “stages”), this critic of comparative deductions relied for the timings on the macaques (Heuser and Streeter 1941, p. 45; Streeter 1942, pp. 213–214; O’Rahilly 1988, pp. 101–106; Hopwood 2005, pp. 281–284) (Fig. 1B).

Clinicians could not treat women like monkeys even then, but emulated the control by exploiting routine operating lists. The anatomist Edgar Allen cooperated to recover unfertilized tubal eggs (Allen et al. 1930). Then, between 1938 and 1954, pathologist Arthur Hertig and gynecologist John Rock of Harvard Medical School took an ethically somewhat perilous approach to retrieve 34 specimens (24 of them normal) through the first two weeks. Rock’s assistant Miriam Menkin had patients waiting for clinically indicated hysterectomies and oophorectomies record their periods. She raised Hertig’s chance of finding embryos at the desired stages by scheduling the procedure accordingly and encouraging the women to have unprotected sex on particular days. With this famous and notorious “egg hunt” the Carnegie Department began to fill the gap at the start of development and offered an influential estimate of the rate of reproductive wastage (Hertig et al. 1956, 1959; McLaughlin 1982, chap. 4; Marsh and Ronner 2008, pp. 92–104; Jarvis 2017).

It might have looked at war’s end as though human embryology would go from strength to strength. The “ovum factory” was “working full-blast.”^8^ Rock and Menkin had just fertilized cultured ovarian eggs in vitro to produce the first human two- and three-cells ever seen (Rock and Menkin 1944; McLaughlin 1982, chap. 5; Marsh and Ronner 2008, pp. 104–110). Streeter was reviewing the collection for his staging project. Results fed into lectures in human embryology that were under pressure but remained fixtures in medical schools (Cummins 1946; Scott 1982). Magazines boasting “amazing photographs” filled gaps in “the oldest human story.”^9^

In fact, research on humans was losing out even at the Carnegie Department. The third director, George Corner, had done morphology himself and followed its progress with pleasure, but this codiscoverer of progesterone wrote of “diminishing returns” (Corner 1941, p. 190). Appointed in 1956, the next director, James Ebert, came from studies of protein synthesis during chick development. He saved the department from closure by refocusing on cellular, molecular, and genetic approaches better pursued in more convenient species. The institute built around human embryos demoted them to an “ancillary position” (Ebert 1957, p. 300; Singer 2004). A decade and a half later, having moved away from the medical school, it “terminated” the program in human embryology (Ebert 1969, p. 501). In 1973, after a deal to bring the “unique,” “magnificent” collection to Wayne State University in Detroit fell through, it went to the University of California, Davis.^10^

At first, with “a delightful setting” near the Primate Research Center, a brave face was put on the move (O’Rahilly and Gardner 1975, p. 97). At the opening curator Ronan O’Rahilly said, defensively: “The collection is not a museum. […] It’s a bureau of human development.” Ebert argued that, as birth defects loomed larger following the decline in neonatal mortality and morbidity from other causes, “the descriptive evidence of human development takes on new importance” (quoted in Econome 1975; on teratology, see Dron 2016).

By 1988, with the embryos shunted out to a “barracks”-like building, the facilities were “by no means ideal.” In an “inadequately ventilated” attic, “practically unbearable in summer,” the cabinets of slides were pushed into “large blocks” and the “small triangular metal work surfaces” in the corners of the room barely took a microscope.^11^ O’Rahilly had extended Streeter’s horizons as the Carnegie stages and, with Fabiola Müller, revised these (O’Rahilly 1973; O’Rahilly and Müller 1987). But a tradition was ending.

Other collections rose and fell on different timescales. In Kyoto from 1961 anatomists interested in teratology collaborated with gynecologists to collect unselected embryos and fetuses under a law that allowed abortions on demand from couples. Hideo Nishimura contested the macaque-based timings of Streeter’s horizons—which O’Rahilly adjusted—and refined estimates of loss rates (Nishimura et al. 1966, 1968; Nishimura 1976; Fannin 2018). But all such repositories soon had their best days behind them.

Announced in the mid-1950s, the making of developmental biology accelerated the reorientation. Inheriting the ethos of experimental embryology, developmental biologists found description worthy but dull. Setting out to embrace the whole living world, they ended up concentrating on a handful of species suited to genetics, micromanipulation, cell biology, biochemistry, and molecular biology. By the 1980s model systems buzzed, croaked, cheeped, and squeaked with excitement. Researchers were combining old and new—genetic screens, gene cloning, monoclonal antibodies, microsurgery, and lineage tracing—to discover the mechanisms that specify body plans (Hopwood 2009, 2019). Surprising evidence of molecular pathways conserved between phyla, in the first place *Hox*-gene specification of the anteroposterior (head–tail) axis, reinforced the assumption that other animals teach most about humans (Gaunt 2019, chaps. 6–8).

The models were to a small extent exemplars of various taxonomic groups and above all surrogates for human beings (Bolker 2009). Jackson Labs having standardized mice genetically, from the late 1950s their conceptuses were opened up to manipulation in culture before transfer to a uterus for implantation, gestation, and birth (Graham 2000; Rader 2004; Myelnikov 2014, chap. 1; 2019). Taking over as the model mammal, by about 1980 they accounted for the most papers on any species published in developmental biology journals (Davies 2007). Their importance was reinforced, though also challenged, by innovation in research on reproduction.

## Live and In Vitro

The culture of human embryos came out of the deeper interventions of science into mammalian reproduction after World War II (Hopwood et al. 2018, part V). Artificial insemination in cattle and funding for population control generated a critical mass of research. To control conception from the female side, an expanding community of agricultural and academic biologists trialled in vitro fertilization and embryo transfer on laboratory mammals. Replacing one-offs with repeatable procedures backed by quantitative evidence, they challenged anatomists’ and gynecologists’ standards while remaining dependent on clinicians for human material. From the Worcester Foundation for Experimental Biology, M. C. Chang’s claim to have fertilized eggs in vitro and produced live rabbits, still the main mammal in studies of reproduction, passed muster under the new criteria in 1959 (Chang 1959; Hopwood 2018a, pp. 587–588).

Slow progress in other organisms, with successes in rodents but failures in livestock and nonhuman primates, highlighted mammalian diversity. The pressures to bypass genetic disease and infertility resulting from blocked Fallopian tubes, as well as some technical advantages, put humans next. The Cambridge geneticist Robert Edwards and Patrick Steptoe, the British laparoscopy pioneer, reported early stages of fertilization in vitro in 1969 (Edwards et al. 1969). Two years later they announced blastocysts, which form when the initial ball of cells, or morula, differentiates into trophectoderm (which makes the placenta) surrounding the inner cell mass (which gives rise to the embryo proper) and a fluid-filled cavity (Steptoe et al. 1971).

Commentators divided over the species politics. Even the supportive *Nature* admitted that, “[f]rom a strictly scientific point of view,” human in vitro fertilization “was merely a repetition for man.”^12^ Chang downplayed it as, “if […] genuine […] perhaps not so extraordinary, since the basic facts are known in other species” (Chang 1971, p. 7). Socially and medically, Edwards was still right: “the switch from animals to humans is an enormous jump” (Edwards 2005, p. 305; Hopwood 2018a, pp. 588–590).

That jump was impossible in the United States. As childbirth moved into hospitals and obstetricians monitored pregnancy more, they had constructed a fetal patient (Arney 1982; Oakley 1984, chap. 7). Following the reform of 19th-century antiabortion laws, a backlash against access to abortion grew through the 1970s. A powerful movement opposed research on human fetuses and embryos and imposed a *de facto* moratorium on federal funding (Maynard-Moody 1995; Dubow 2011, pp. 75–79; Hurlbut 2017).

In the United Kingdom the Medical Research Council (MRC) rejected a grant application from Edwards and Steptoe in 1971. The referees had ethical reservations about transferring blastocysts to patients and wanted experiments on nonhuman primates first (Johnson et al. 2010). But NHS support, money from a US TV-station heiress, and ethical persuasion let Steptoe and Edwards perform hundreds of transfers on volunteers over many years of trial and error. They announced a birth in 1978 and another in 1979 (Johnson and Elder 2015). While few had noticed Chang’s rabbits, the “test-tube baby” made global news and a medical industry (Hopwood 2018a, pp. 590–595; 2018b, pp. 650–654; 2018c; Marsh and Ronner 2019; Ferber et al. 2020).

The MRC converted to strong support for research, especially through grants to Edwards’s former student Martin Johnson and clinician Peter Braude in Cambridge. Scientists, physicians, and affected families campaigned for research on embryos not used for reproduction (Mulkay 1997). As the debate heated up *Nature* opined:

The extent to which knowledge of what happens—and what goes wrong—in the early stages of natural embryonic growth can be extrapolated from observations of other than human mammals is strictly limited. […] And one of the next big prizes in biology, an understanding of the process of differentiation […] will in due course probably require confirmation in human embryos grown beyond the gastrulation stage (two to two-and-a-half weeks). When that time comes, the potential benefits in medicine are also likely to seem overwhelming.^13^

When researchers realized what a mountain of skepticism they would have to climb, they underlined medical benefits, not prizes in biology.

In 1984 the UK government committee of inquiry chaired by the moral philosopher Mary Warnock recommended the licensing of research up to 14 days after fertilization—a somewhat arbitrary limit presented as coinciding with the acquisition of individuality or “when I began being me” (McLaren 1984, pp. 109–112). The majority argued that “in certain situations there is no substitute for the use of human embryos.”^14^ Experimenting on nonhuman animals was controversial in the run-up to the Animals (Scientific Procedures) Act 1986, but perfecting techniques on them meant that “the information peculiar to the human can be obtained from relatively small numbers of eggs and embryos.”^15^ Critics, mainly antiabortionists and including a minority on the Warnock Committee, presented research on human embryos as unnecessary. Animal experiments and innovation during medical treatment supposedly sufficed.^16^

Between evidence of overwhelming parliamentary opposition in 1984 and legislation in 1990, UK researchers lobbied for a more congenial regime. Factsheets rebutted the claim that only animals were needed: humans have “very different reproductive systems,” with different genetic diseases and repeated miscarriages more common, they argued.^17^ Medical bodies also prefigured regulation with a Voluntary (later Interim) Licensing Authority. Applicants had to “give reasons why information cannot be obtained from studies of species other than the human” and to define an aim “relevant to clinical problems such as the diagnosis and treatment of infertility or of genetic disorders” or better contraceptives (quoted in Gunning and English 1993, p. 186).

**Fig. 2:**
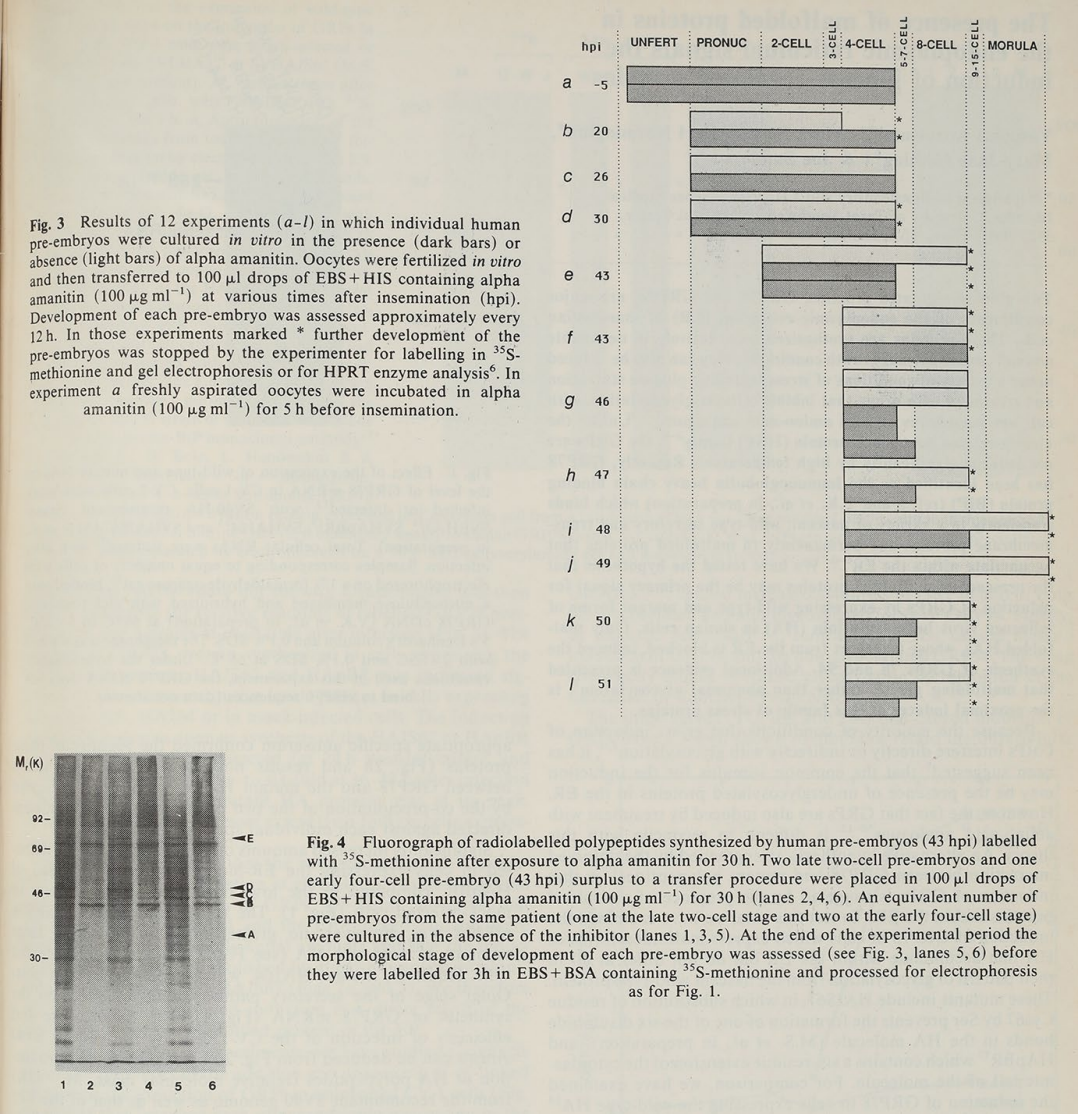
Braude et al.’s analysis of development and polypeptide synthesis in individual human conceptuses in the presence (fig. 3: dark bars) or absence (light bars) of the transcriptional inhibitor α-amanitin. This affected development beyond but not up to the four-cell stage, with apparent exceptions put down to the paucity, variable quality, and doubtful normality of material, mostly “spares” from an IVF program. The bands labeled with ^35^S-methionine in fluorographs of 1-D polyacrylamide gel electrophoresis (fig. 4) approximated a “late” pattern in untreated conceptuses (lanes 1, 3, 5) but stayed in an “early” one in the treated (lanes 2, 4, 6). From Braude et al. (1988, figs. 3–4), with permission of Springer Nature

Campaigners won public support and divided the opposition by accomplishing preimplantation genetic diagnosis based on amplifying Y-chromosome-specific DNA from biopsied cells to select female embryos and so avoid X-linked conditions (Franklin and Roberts 2006, pp. 44–60; Theodosiou and Johnson 2011). In the run-up to this success, MRC units tried other methods, and in particular promoted work on mice and humans through Lesch-Nyhan disease, a rare but serious X-linked disorder of purine metabolism. At the Mammalian Development Unit in London Marilyn Monk learned to test biopsied mouse embryos for activity of the affected enzyme, hypoxanthine-guanine phosphoribosyl transferase (HPRT). The Cambridge group adapted the assay for humans but showed more generally by radiolabeling proteins and inhibiting transcription that the zygotic genome was not activated until the four- to eight-cell stage rather than at two to four cells as in mice (Braude et al. 1988, 1989) (Fig. 2). To detect embryonic (not maternal) enzyme, cells would have to be removed later.^18^

As part of their battle for research on human material, Braude and Johnson mobilized these results, which

clearly demonstrate that mouse and human eggs and pre-embryos [conceptuses up to 14 days] are sufficiently close for work on mice to provide important basic information, helping focus […] questions and refine technology […]. However, it is also clear that the direct therapeutic application of technology developed on the mouse to the human will not work. The human pre-embryo is sufficiently different to make a further limited period of experimental research directly on the human conceptus required. The choice before Parliament […] is whether that research will be permitted until such time as the transfer of technology has been achieved.^19^

The commitment to a model organism together with insistence on human work, but of restricted scope, is typical. Within that framework, the emphasis ranged from the need for confirmation—results “must be checked on human material” before these “can be put into clinical practice”—to vivid examples of “[t]he dangers of extrapolating from mouse to human” (McLaren 1986; Vines 1989).

The Human Fertilisation and Embryology Act 1990 enshrined a permissive but regulated regime, with a 14-day limit, that made the United Kingdom a center for the science. Research to “increase knowledge about […] development” still came after a long list of clinical purposes.^20^ Subject to various constraints, human embryo research was allowed also in other jurisdictions.

On the one hand, preimplantation stages, long almost impossible to obtain, were now accessible through arrangements between laboratories and clinics. Atlases charted development from oocyte to blastocyst in unprecedented detail (Veeck 1986; Magli et al. 2012). Expression studies of individual genes, like cDNA libraries from single embryos, relied on reverse transcription followed by the polymerase chain reaction to amplify the minuscule amounts of DNA. Researchers claimed that “the biology of very early human development is establishing its own legitimacy” but dwelled on “significant deficiencies” (Pergament and Fiddler 1998).

For, on the other hand, biologists were frustrated that clinics froze hundreds of thousands of embryos not destined for reproduction (which took the best), while few and often abnormal specimens came their way (even though more clients would have liked to donate). Scarcity and variability exacerbated the challenges of biochemistry or molecular biology on tiny eggs. Academics disparaged the preponderance of low-quality clinical studies and the adoption with insufficient evidence of procedures to raise low rates of pregnancy following IVF. They lamented slow progress through the 1990s (Van Blerkom 1994).

Scientists’ mobilization behind in vitro fertilization had established research on living human preimplantation embryos and would lead to further innovation, in part via stem cells derived from them. But the larger revival did not come only out of IVF or apply just to the earliest stages. Next curators reanimated anatomy by digitizing slides in a museum.

## Visible Embryo Projects

“Changes in medical education over the past 3 decades have not been kind to embryology courses,” an Oxford anatomist wrote in 1998. Compared with the excitement of reproductive biomedicine, medical students could find descriptive human embryology dull—even when their teachers added congenital malformations and mechanisms discovered in model organisms (Morriss-Kay 1998). But lectures were still given and developmental anatomy gained research potential through the 1990s. Gene expression would be mapped using structural knowledge revised as well as remediated for the digital age. Large existing collections represented stages better than any new venture. Their ethical use was usually easier.

Preparing for O’Rahilly’s retirement in 1990, the Carnegie Institution invited bids to house the collection. Having experienced two failures of universities to provide long-term homes, he insisted on a national (or international) body. In 1991 the embryos, models, and documentation arrived at the National Museum of Health and Medicine of the Armed Forces Institute of Pathology (AFIP) at the Walter Reed Army Medical Center in Washington, DC, which secured them in a building designed to resist a nuclear blast.^21^ Museum assistant director Adrianne Noe and AFIP deputy director Colonel Thomas Stocker set up a Human Developmental Anatomy Center (HDAC) to serve as “the nucleus” for collaboration with researchers and “a magnet” for other collections.^22^ Lower-key than the Visible Human Project® with its adult male and female cadavers in cyberspace (Cartwright 1998; Kasics 2000; Waldby 2000), “visible embryo” projects rode a wave of revolution in medical communication.

“Computer archiv[ing] and modeling,” as initially presented by anatomists Don Hilbelink (University of South Florida, Tampa) and Raymond Gasser (Louisiana State University Health Sciences Center, New Orleans), aimed to overcome “geographical and/or economic constraints” on use (Hilbelink and Gasser 1990). To this they soon added, as HDAC recruited them, an urgent need to preserve the information on aging slides.^23^ In time Gasser also argued that it was quicker to locate sections in a virtual model than on slides; that labeling helped nonanatomists; and that connected, quantitative data about the relative positions of structures would fill gaps and correct errors springing from partial views. Having envisaged use to assess malformed morphology, project directors increasingly provided a structural framework for studies of gene expression.^24^

Other species were mentioned here only in the old war dance against errors of inference. “Much of what’s in the textbooks was extrapolated from animals and is sometimes wrong,” researchers said (quoted in Leslie 2003), as they promised the rebirth of human embryology (Gasser et al. 2014). Elsewhere, living preimplantation embryos offered virgin soil. Here a neglected field of work on postimplantation material, variously overgrown, polluted, and barren, was to be made cultivable again.

In 1992–1995 the National Institutes of Health (NIH) issued HDAC with three one-year contracts to inventorize the Carnegie collection, capture digital images of the serial sections of selected embryos, and visualize identified organ systems on optical discs. Sponsored by the National Institute of Child Health and Human Development (NICHD), the Office for Research in Women’s Health, and the National Center for Research Resources, these datasets would “serve as a springboard for the investigation of super-computer-based, image-reconstruction processes and for cooperation with institutions using wideband-width image transmission technologies.”^25^

For five embryos of different stages, HDAC imaging specialists Elizabeth Lockett and William Discher worked with Gasser, who had trained with the controversial German anatomist Erich Blechschmidt and authored an atlas.^26^ The team took digital photos of sections, generated contours of anatomical domains, transmitted these to a computer, and converted the contour files into virtual, rotatable 3D models (Noe 1996; further: Doyle et al. 1996). Clicking on a website brought up photos of the sections and short movies, including flythroughs.

Further funding entered Carnegie embryos into a competition among new imaging modalities. With $1M from NICHD in 1996–2001, Bradley Smith (Duke University then University of Michigan) scanned 14 whole preparations nondestructively with magnetic resonance microscopy (MRM). He assembled the slices into virtual models, “segmented” these, that is, manually identified contours of organ systems, and annotated them (Smith et al. 1999; Smith 1999; Morgan 2009, pp. 208–212). MRM images featured in an exhibition and a coffee-table book advertised on *Oprah*.^27^ The technique was slow and the resolution rather low. Kyoto anatomists used more powerful magnets and a “super-parallel” microscope to see more and faster, but MRM did not catch on (Shiota et al. 2007).

**Fig. 3:**
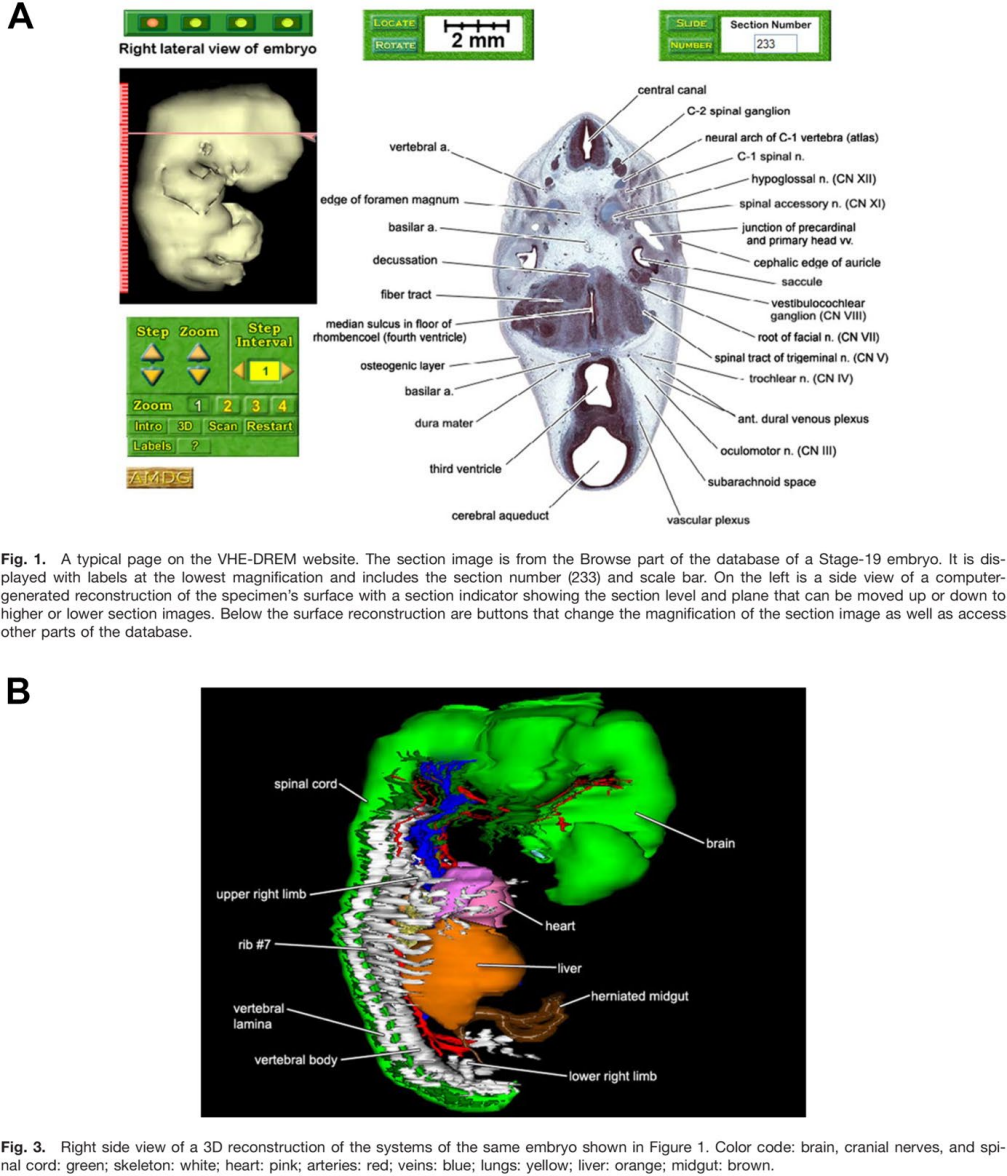
Gasser demonstrates his Visible Human Embryo–Digitally Reproduced Embryonic Morphology website on a Carnegie Stage 19 embryo. **A** Section selection, with structures on this one labeled. **B** Three-dimensional reconstruction of the anatomical systems. Gasser et al. (2014, figs. 1 and 3). © 2014 Wiley Periodicals, Inc.

HDAC had proved the digitization concept on a few embryos requiring massive datasets shared over sluggish network connections. In 1999 it joined the Next Generation Internet Initiative of the National Library of Medicine (NLM).^28^ For seven years from 2000 Gasser and software developer John Cork used $3M from NICHD to produce a teaching resource of sectional photos, with structures segmented and labeled, surface reconstructions, and volumetric models for all 23 Carnegie stages (Fig. 3).^29^ They distributed this Virtual Human Embryo on DVDs. The NLM gave $0.6M for a web interface that allows searching by anatomical terms. Most users access it through the site of the Endowment for Human Development, an educational nonprofit with a policy of bioethical neutrality. University teaching and pregnancy apps incorporated the data.^30^

Digitization went international. Between 2009 and 2016 anatomists in Amsterdam produced an atlas for medical education and to provide a baseline against which to identify congenital abnormalities. They bypassed an alleged mess of unverifiable or inaccurate textbook diagrams by reconnecting with original slides: those photographed for Gasser and another series, mostly from the Carnegie Collection, to reduce potential distortion by individual anomalies. In an impressive investment of time, 75 students contributed 45,000 hours of labor to segment the structures in 15,000 sections. These were virtually reconstructed in 3D and the surfaces smoothed. Published in *Science* magazine, interactive 3D-pdfs let a user rotate the models on a laptop or phone and select labeled structures in bright colors (De Bakker et al. 2016).

Project co-leader Bernadette de Bakker told *The Guardian* newspaper, “Everyone thinks we already know this, but […] we know more about the moon” (Davis 2016). Elsewhere De Bakker presented publications as accurate until around 1980, when results from nonhuman animals contaminated the textbooks (De Bakker et al. 2012, p. 234), for example, concerning the notochord (De Bakker et al. 2016). To distinguish the reliable knowledge, the anatomy had to be digitally redone.

The Kyoto center and then German groups joined in (Yamada et al. 2006, 2012; Brand-Saberi et al. 2012). A Digital Embryology Consortium, established in 2014, shared an automatic scanner as partners enrolled (Hill 2019). Amidst a general reappraisal of anatomical collections, curators faced questions about the provenance of embryos and fetuses collected without patient consent. Most troublingly, Erich Blechschmidt’s right-wing Catholic politics (antievolutionist as well as antiabortionist) attracted accusations that, as a professor in Göttingen since 1942, he had stockpiled material from forced abortions and sterilizations under National Socialism. A recent inquiry found no evidence of the deliberate exploitation of such practices, but the unprofessionally sparse records could not exclude sourcing from contexts of injustice (Markert 2019, 2021).

Websites provided community resources. Mark Hill, an anatomist at the University of New South Wales, curates the closest approach to a single portal such as FlyBase for *Drosophila*. This began with students labeling images of embryonic histology. In 1997 Hill put it on the Internet, where it gives access to a rich timeline of development and thence to classical publications and current projects. A second site leads to the digitized images from the consortium.^31^ Their importance is illustrated by citations and the fact that anatomists still travel to undigitized embryos. The uses of what came to be called “archival” collections are limited nevertheless.

## Biobanking Postimplantation Embryos

Rising to dominate studies of development during the 1980s, molecular analyses of gene expression needed fresh or promptly frozen preparations. Clinics had traditionally provided postimplantation material from terminations of pregnancy through arrangements with individual laboratories and central collections. “Biobanking” would transform the logistics. As in Baltimore and Kyoto, biobanks handled collection from various sources according to the prevailing regulations. Unlike the central collections, banks distributed the material. Researchers profited from the “ethical efficiency” of preparations cleared for general use.^32^

Coordinated collecting and distribution began in the United States with teratologist Thomas Shepard’s Laboratory for the Study of Embryology (later Laboratory of Developmental Biology) in liberal Seattle, which the NIHCD has funded from 1966 (Shepard et al. 1988). Abortion-law reform made more fetal tissue available for research and therapy, principally transplantations to alleviate Parkinson’s disease, but antiabortionism constricted the supply (Casper 1994; Maynard-Moody 1995). In the United Kingdom from 1957 to 2004 the MRC supported a Fetal Tissue Bank at the Royal Marsden Hospital, London, with developmental biologists among the recipients (Gal 1973; Lawler 1981). Compared with preimplantation embryos controversy was slight, and the 1989 Polkinghorne Report set out a safeguard: the decision to terminate should be separate from consent to donation and clinic staff not stand to benefit directly.^33^

Though the usual methods of termination damaged embryos and fetuses, skilled gynecologists could recover these intact. For the Kyoto collection they had performed careful dilation and curettage (Nishimura et al. 1968), but when vacuum aspiration became standard it was even harder to remove whole fetuses. Ultrasound guidance enabled this up to ten weeks of gestation, although with more fragmentation at later stages, as Charles Rodeck showed at London’s University College Hospital (UCH) (Soothill and Rodeck 1994). Medical abortion with mifepristone (RU-486) and a prostaglandin provided younger embryos, a higher proportion of them intact (Bullen et al. 1998). A group at the Necker children’s hospital in Paris used a series to study *PAX* (paired-box transcription factor) gene expression and followed similar ethical protocols (Gérard et al. 1995).

In 1994 fetal medicine units in London and Newcastle obtained ethical approval and began supplying. Rodeck collaborated with developmental biologist Peter Thorogood at the Institute of Child Health (ICH) and Stephen Robson with molecular geneticist Tom Strachan at the University of Newcastle. After local funding at each site, the Wellcome Trust awarded Newcastle a three-year project grant from 1996. The MRC similarly backed the London bank, which started to send first-trimester material around the country. In 1999 parallel MRC and Wellcome grants supported the integration of the centers into a collaborative Human Developmental Biology Resource (HDBR) with a joint steering committee and harmonized records.^34^ When Thorogood died suddenly in 1998, developmental biologist Andrew Copp took over at ICH. Susan Lindsay worked with Strachan in the Newcastle Department, later Institute, of Human Genetics. A series of joint MRC–Wellcome five-year grants rose from a total of £1M for 2002–8 to £5M for 2023–27.^35^

With collection from terminations potentially contentious, announcements were limited, even within biomedicine.^36^ The funders helped choose a name to avoid the controversy associated with abortion and “embryo research.” As Lindsay and Copp recalled, “Developmental Biology Resource: it’s not obvious what it is really […] I’m sure that was part of the thinking.” “I think there was […] concern that […] there might be a lot of antagonism from […] antiabortion groups […] so perhaps this title was chosen as being the least […] controversial [and most] academic.”^37^ Controversy avoided, the resource was advertised more.^38^ The name also recognized that “human embryology” now referred to work on preimplantation stages, particularly the procedures that clinical embryologists perform for IVF. Researchers who might otherwise have kept the traditional identity called themselves “developmental biologists” instead.^39^

Most material came from Newcastle-area hospitals and UCH and then also British Pregnancy Advisory Service clinics. Having two regional hubs with different profiles—mainly medical terminations in Newcastle and surgical in London—“buffered” the unpredictable supply to the HDBR.^40^ The 2020 coronavirus pandemic revealed the vulnerability of that supply when UK guidelines were relaxed to allow medical abortion at home (a widely welcomed move) and the plummeting availability of earlier stages created “a disaster” for the HDBR.^41^ Since then, it has trialled “research clinics” for those who prefer medical termination in a hospital. Many donate.^42^

The HDBR distributed material and pushed, with growing success, for the results to be deposited in databases. It has prioritized projects by relevance to human disease, novelty, and user competence.^43^ Network members receive preparations fresh, fixed, or rapidly frozen to preserve the delicate RNA. From 2004 they could use an in-house gene expression service.^44^ Much of the material was karyotyped for reassurance about normality. The HDBR also stocks abnormal samples and has expanded to collect from clinics that perform terminations for fetal anomalies.^45^

As a result of innovations I shall describe, demand took off around 2015 in terms of numbers of projects (some 275 in 2018–2021), tissue samples provided (nearly 13,000), and publications that included data derived from HDBR material (over 30 in 2021).^46^ In 2000–5 almost all registered projects were UK-based, with fewer than 7% in France, Germany, Italy, and the United States combined. Between 2012 and 2017 20–37% of annual registrations came from teams in 17 foreign countries.^47^ The Seattle laboratory has survived antiabortionist activism, including an undercover-video scandal in 2015 and hostile policies under the Trump administration. Similar biobanks were set up in the Netherlands in 2017 and France in 2020. Laboratories continue to make individual arrangements with clinics.^48^

Still the largest and most international resource, the HDBR has supported various kinds of study, but primarily of gene expression. For this purpose Strachan’s group originally pressed the case for funding and advocated for human work.

## Gene Expression That Makes Us Not Mice

Through the Human Genome Project (HGP) of the 1990s research into gene expression as a clue to function leant on an improved mouse model and was valorized as going beyond it. This argument drove the establishment of the HDBR.

As the HGP bureaucratized “model organisms” and sequenced the mouse genome—a draft came out in 2002—medical demands favored this rodent as the model human.^49^ Skills and knowledge having flowed from fruit flies and lower vertebrates, the NIH and other funders exploited the ability to generate germline mutations, not least to model human diseases. International programs assessed the phenotypes of thousands of knockouts (Adams et al. 2013; Davies 2013). With MRC support, anatomist Matthew Kaufman and his Edinburgh colleagues collaborated with Jackson Labs to build, in part from photos of sections in his developmental atlas, a series of electronic 3D models in which to lodge expression data—a digital and molecular updating of slice, stain, and reconstruct (Baldock et al. 1992; Richardson et al. 2014).

**Fig. 4:**
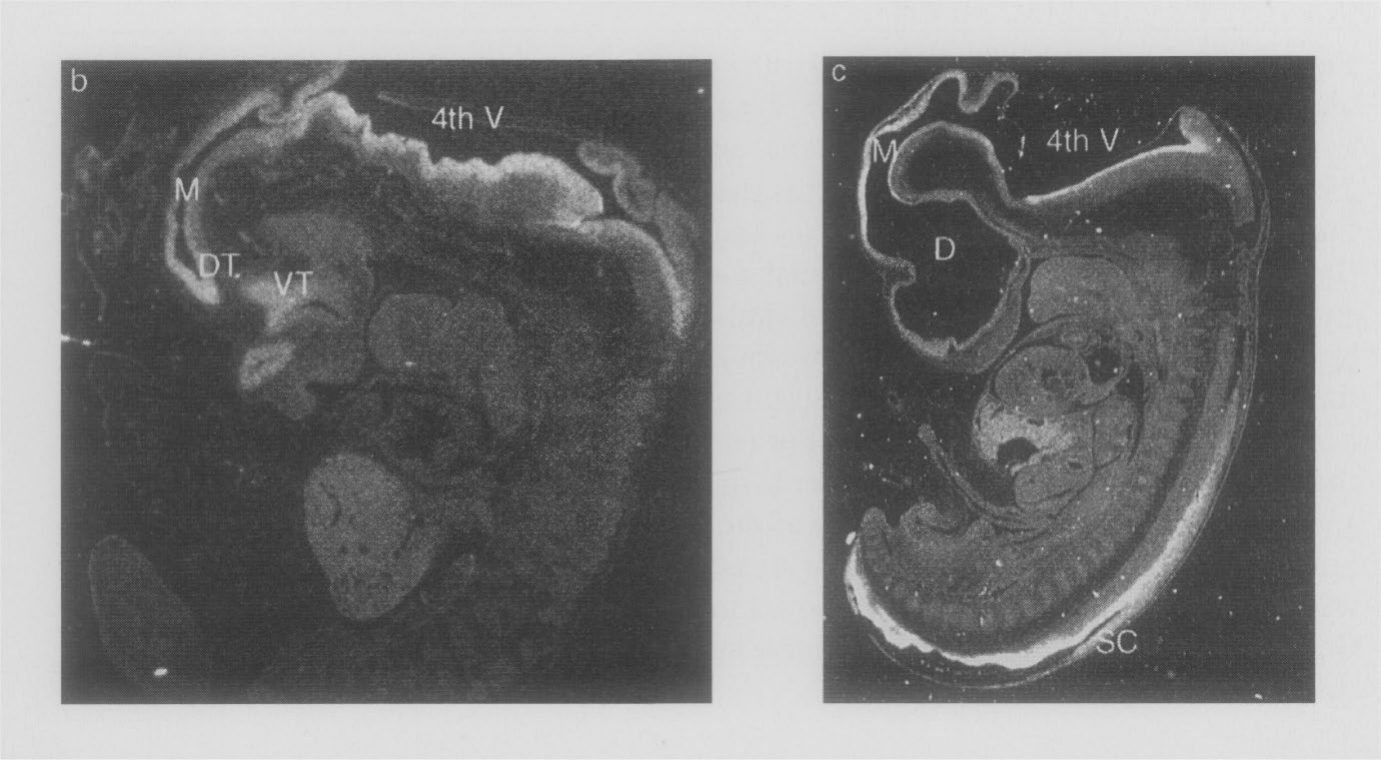
Dark-field photomicrographs of in situ hybridizations of sections of Carnegie Stage 15 and 17 human embryos in the landmark book, *Molecular genetics of early human development*. ^35^S-labeled antisense RNA probe for *WNT7A* produced silver grains in the emulsion that show expression in the developing brain. From Lindsay et al. (1997, p. 206), with permission from Elsevier

The improving mouse model moved scientists to seek the similar and the different in material of supreme clinical and anthropological relevance. Led by Strachan, who had studied gene families and was mapping disease loci, the Newcastle geneticists argued that, as the HGP identified genes, understanding their functions and malfunctions needed information about expression through human embryogenesis. In situ hybridization (to detect mRNA) and immunocytochemistry (using antibodies against proteins) must be pursued systematically, like for the mouse, with an infrastructure to share a precious resource and pool results (Fig. 4).^50^

Yet conclusions such as this one, in a 1997 paper from Strachan’s lab about a gene encoding a transcription factor involved in forelimb and heart development, would comfort investors in the mouse model: “The *TBX5* expression pattern in human embryos is very similar to that reported for *Tbx5* in mice. It has not been possible to establish a complete temporospatial human expression pattern due to the limited availability of human embryos” (Li et al. 1997, p. 26). A referee of a Wellcome Trust application objected, though this was a minority view: “Why bother studying gene expression in human embryos when they are difficult to obtain and cannot tell us anything we could not learn from studying plentiful mouse embryos?”^51^

In response, Strachan and colleagues highlighted the recency of developmental biologists’ conversion, following the discovery of *Hox* genes in the 1980s, to assuming conservation of processes across the animal kingdom. Human and mouse might look “profoundly similar” at early stages—though, in fact, we develop, like most mammals, as a disc, and rodents, unusually, as a cylinder. But the two mammals’ last common ancestor lived 80 million years ago and the adults were “remarkably dissimilar.” No wonder some human genetic conditions could not be modeled; knocking out the *HPRT* gene in mice failed to cause a condition like Lesch-Nyhan disease. Some genes lacked counterparts in mice, some orthologs diverged, and others were differently expressed (Burn and Strachan 1995, pp. 4, 6).

Strachan and colleagues turned these disappointing results into an opportunity to study what “makes us unique, or at least not mice” (Burn and Strachan 1995, p. 6). Building on the medical-benefit case for investigating preimplantation embryos, but relying on established uses of abortus material and the relevance of the stages of organogenesis to brain development and congenital disorders, they articulated a more confident rationale. This targeted “human consciousness,” so that “we might finally be able to answer such questions as: *why are we able to ask questions?*” (Burn and Strachan 1995, p. 6; italics in the original). They recognized that, given the ethical constraints, “the human will never challenge the mouse […] in experimental embryology.” But “the human embryo should” not “be relegated to the rather tranquil scientific backwater of comparative mammalian embryology.” Scientists knew “a large number of naturally occurring, well-studied developmental pathologies” in our own species which should be researched because humans are different and in the hope of preventing disease (FitzPatrick 1997, p. 10).

Rejecting extrapolation, the Newcastle group constructed a flexible argument. Finding the same gene expression gave “very important” reassurance about the applicability of the model, while divergent patterns pointed to functional differences and so were “very important” to follow up (Lindsay et al. 1997, p. 209). The win-win reasoning kept the mouse, as Strachan and Lindsay put it, an “extremely valuable” but essentially “imperfect” model (Strachan and Lindsay 1997, p. 14). It was not the only one—prioritizing tractability, some modeled human diseases in flies (Anderson and Ingham 2003)—but the mouse–human dyad has structured the field.

The argument met “vested interests” in mice and “disbelief.” A colleague of Lindsay was explicating a poster at the 2007 meeting of the European Society of Human Genetics, when the visitor “suddenly looked at her and said, ‘But you don’t mean that that’s a human embryo? […] I just assumed it was mouse.’”^52^ The work faced accusations, of the kind leveled at genomics in general, of “stamp collecting” or lacking hypotheses. As Lindsay recalled, “developmental biologists from a whole range of animal model systems were happily wanting to explain to us that we couldn’t do experiments with our material.” “[W]e had to keep banging this drum for really quite a long time.”^53^ Wellcome and MRC had heard the drum and funded the HDBR. This broadcast the beat by sharing stocks of human embryos while demand grew and, thanks to postgenomic methods and new imaging methods, doable studies gained in interest and status.

To create a community resource, the group followed the Edinburgh mouse researchers by preparing virtual 3D models of staged embryos into which data would slot. Optical projection tomography, an imaging technique invented in the Scottish capital, gave higher resolution than MRM and could handle larger specimens than confocal microscopy. Projects, mainly funded by NIH and about the brain, were consolidated by 2010 in an atlas the HDBR took over and generalized.^54^

**Fig. 5:**
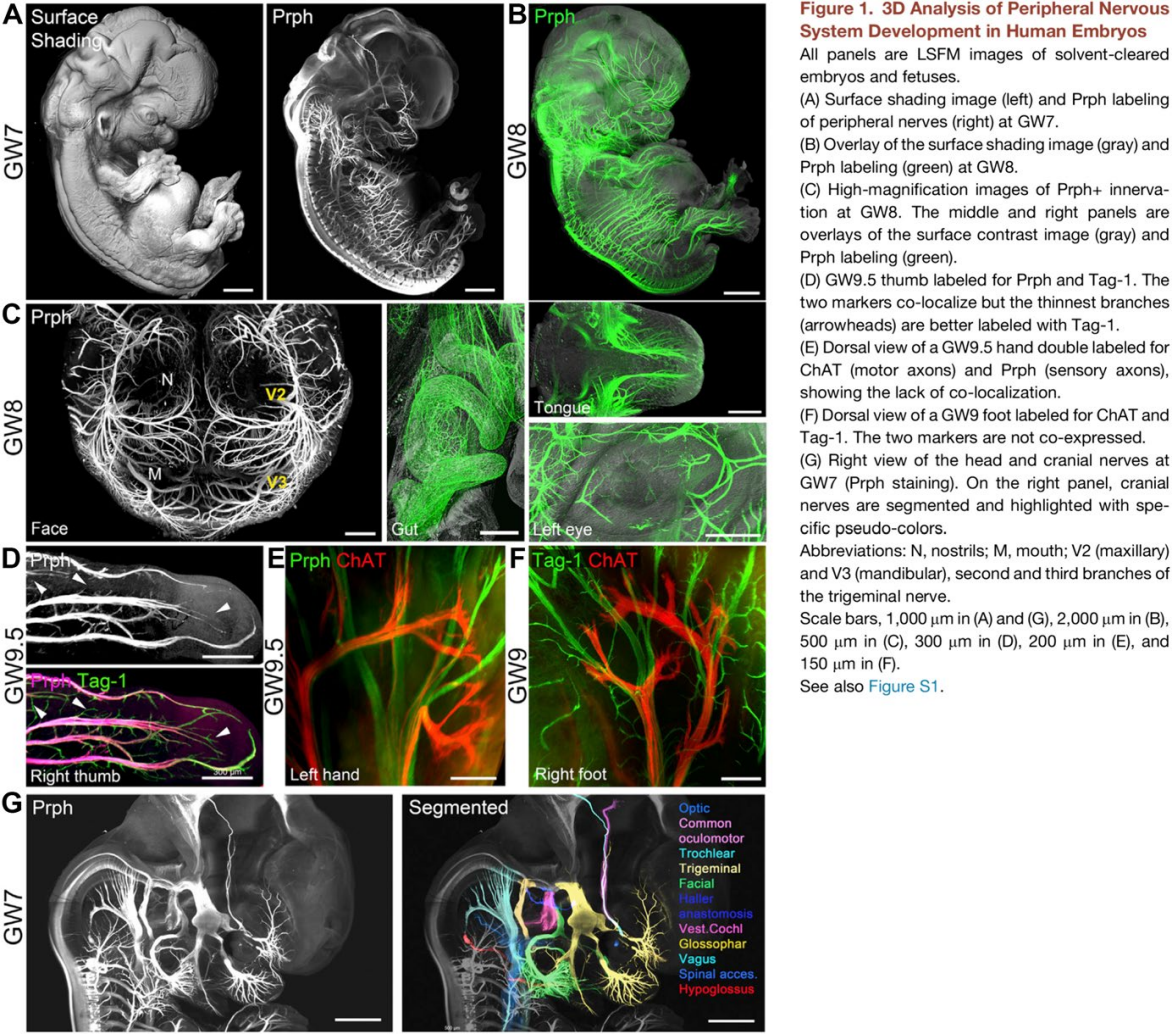
3D analysis of peripheral nervous system development in light-sheet fluorescence microscope images of human embryos (gestation week 7 to 9.5) cleared and stained with antibodies (Prph, ChAT, Tag-1) for neuron-specific proteins in Alain Chédotal’s lab. From Belle et al. (2017, fig. 1), with permission from Elsevier

An imaging revolution proceeded apace, refining digital anatomies and spotlighting gene expression. Confocal laser scanning microscopes optically sectioned cleared specimens with minimal background light for assembly into 3D images. From around 2014 light sheet fluorescence microscopy caused less bleaching and damage, letting samples be imaged for longer. Alain Chédotal’s lab at the Vision Institute, Paris, stained embryos from medical terminations with fluorescent antibodies against tissue-specific proteins, cleared them, and scanned serial sections. Computer-graphics tools assembled the huge volume of data, then bespoke systems let researchers segment and label anatomical domains (Belle et al. 2017) (Fig. 5). Put on an Oculus headset and you zoom in virtual reality through dozens of identified regions. Pick up a 3D-printed model and you return to physical engagement with developing form.^55^ Pixels and plastic have replaced wax plates and plaster. Adding precision to anatomies based on archival collections, they are correcting long-held views and fleshing out knowledge.

During the 2010s research using “omics,” high-throughput methods that collectively characterize various classes of biomolecules, such as the full range of mRNA, grew to rival expression studies of one or several genes. Scrutinizing whole transcriptomes in tiny samples of embryo tissue, human developmental biologists pursued RNA sequences and chromatin accessibility in single cells (Cao et al. 2020; Domcke et al. 2020). A single-cell RNA-seq analysis of a very early—still gastrulating—preparation obtained through the HDBR serves as a reference. The conclusion is typical: “Comparison with mouse gastrula transcriptomes revealed many commonalities between the human and mouse but also several key differences.”^56^

Such statements can test readers’ patience with the win-win argument while we wait to discover which similarities and differences are really worth knowing. But some of the molecular distinctions being added to deepening comparisons of morphology and timing will be involved in making us not mice. The same logic continued also to justify research on earlier stages that, being alive, attracted more attention.

## Culture, Omics, and Editing; or, “It’s Really Embarrassing”

While funders invested in infrastructure for postimplantation embryos, biologists vented frustration that for lack of material they still barely knew the cellular or molecular mechanisms of preimplantation development. Fertility clinics produced hundreds of thousands of embryos every year that did not go for reproduction. But the vast majority were unavailable for research. Cumbersome regulations, commercial priorities, and underfunded public services meant few biobanks and thus bioethical inefficiency: relationships had to be nurtured with individual clinics and patient consent obtained for specific projects.^57^ Enough material was supplied that new techniques of culture and manipulation, transgressive when applied to humans, could raise the profile of the field.

The making of embryonic stem (ES) cells, which can divide indefinitely and differentiate into any cell type, changed things to an extent. Following work in mice on pluripotent cells from germ-cell tumors, ES cells were derived in 1981 from preimplantation embryos (Evans and Kaufman 1981; Martin 1981; Solter 2006; Maehle 2011; Myelnikov 2014). In 1998 the same team at the Wisconsin Regional Primate Research Center that had made ES cells from macaques created human (hES) lines (Thomson et al. 1998). Another, with experience in mice, derived these from the gonads of human embryos from terminations five to nine weeks postfertilization (Shamblott et al. 1998). Outsized promises depended on, and perhaps helped to grow, the “embryo supply” (Franklin 2006; Franklin et al. 2008). From 2007 a shift began to inducing or “reprogramming” stem cells from adult human skin fibroblasts. Shinya Yamanaka’s group in Kyoto, which achieved this first (Takahashi et al. 2007), had reported the initial breakthrough with mice the year before.^58^

**Fig. 6:**
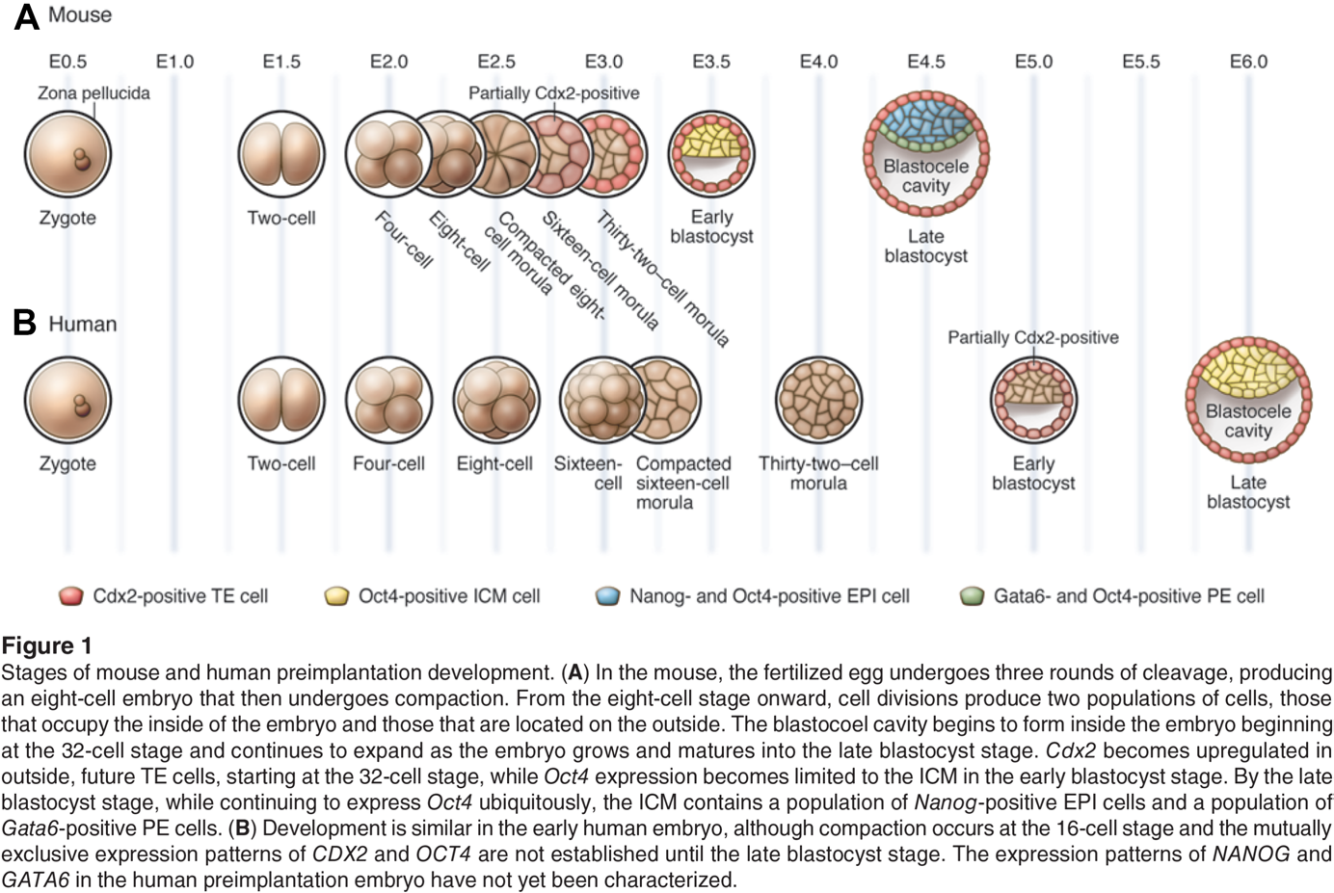
Diagrams in a 2010 review article of stages of mouse and human preimplantation development with colors marking the expression of transcription factors believed to control cell-lineage specification. Ordering by embryonic days (E0.5–E6.0) makes clear the slower pace of human development; ordering by stage aligns processes that take different lengths of time (e.g., Shahbazi 2020, p. 3). From Cockburn and Rossant (2010, fig. 1), with permission of the American Society for Clinical Investigation conveyed through Copyright Clearance Center, Inc.

Also in the 2000s mouse developmental biologists provided imaging, molecular, and omics methods to copy, and results to compare, as they reconstructed the mechanisms that establish the earliest cell lineages. Processes included the separation of the inner cell mass, which gives rise to the epiblast and thence the embryo proper, from two extraembryonic tissues, the primitive endoderm, which forms the yolk sac, and the trophectoderm, which becomes the placenta (Cockburn and Rossant 2010). Researchers discovered differences between humans and mice not just in the timing of zygotic transcriptional activation, but also in gene expression patterns, epigenetic modifications, and aneuploidy rates. They stressed the known inefficiency of human reproduction (Dobson et al. 2004; Zhang et al. 2009; Vassena et al. 2011; Niakan et al. 2012; Niakan and Eggan 2013). Diagrams in papers and talks lined up human and mouse (Fig. 6). They represent the dependence of the human research on the model and the acceptance of subtle similarities and disparities as worth reporting.

The scarcity of the human material reinforced the legal and moral imperatives to use precious samples well. Taken from random populations and patients of infertility clinics, these were also more variable (and representative) than those from inbred mice. Researchers tried to have everything working optimally before thawing them out. At the Crick Institute, London, for example, even the Human Embryo and Stem Cell Lab, where Kathy Niakan focused on translating techniques to work with human conceptuses, spent much time honing the “perfect tools” in mice.^59^ The promise was to understand human specificity with a view eventually to advancing fertility treatment and stem-cell derivation and even maternal and newborn health.

During the 2010s technology transfers from mice to humans made news. Germline genome editing by CRISPR-Cas9 superseded knockouts in mice but was still almost impossible to replicate in human embryos, whether to test function or to act as reporters. In a proof-of-principle experiment, licensed by the Human Fertilisation and Embryology Authority, Niakan’s lab showed in 2017 that removing a transcription factor affected early development more severely in humans than mice (Fogarty et al. 2017; further: Stamatiadis et al. 2021). The barriers to correcting human disease mutations remain high.

Length of culture was another frontier—technical given the need in vivo for implantation, and ethical, because of the 14-day rule. Interactions between embryo and uterine lining had been analysed in vitro but later stages hardly explored (Weimar et al. 2013). Drawing on a system that Magdalena Zernicka-Goetz’s Cambridge laboratory made for mice, in 2016 her group and Ali Hemmati-Brivanlou’s at the Rockefeller University in New York cultured IVF embryos in an oxygenated gel without uterine cells for 12–13 days after fertilization, close to the legal limit. They stained with lineage markers and compared with Carnegie stages to argue that the remarkable degree of self-organization was specifically human (Deglincerti et al. 2016; Shahbazi et al. 2016; Zernicka-Goetz and Highfield 2020, chap. 6). Colleagues worried about inefficient culture and disorganized structures but expected improvements, hoped for better from monkeys, or went back to co-culture with endometrial cells.

Leading figures called for culture beyond 14 days and played up the species politics. Zernicka-Goetz (her output mostly about mice) said: “You have to study the human embryo to understand the human embryo” (quoted in Monahan 2016). Brivanlou (who made his name with research on *Xenopus*) said: “It’s really embarrassing […] that we know more about fish and mice and frogs than […] about ourselves” (quoted in Reardon 2016). In a sign of confidence compared with the 1980s his “rationale for human embryological studies” listed “understanding […] our […] origins” and “investigating species-specific […] differences” ahead of improving reproductive medicine and developing therapies. He admitted that, for securing “support and funding […] utilitarian arguments […] tend to be more effective” (National Academies of Sciences, Engineering, and Medicine 2020, pp. 51, 55, 103).

The species politics are also changing in another way. Although almost every advance in humans has been made first in mice, recognition of the peculiarity of mouse development is making a little space for other mammals. Cynomolgus macaques and marmosets compete as primates that allow (harder and more controversial) experiments. A human-centered renewal of comparative embryology aligns datasets with those from various species in the hope that comparison will decide between ancestral and derived characters (Boroviak et al. 2018; Gerri et al. 2020; Nakamura et al. 2021).

Perhaps because analyses of embryos before and after implantation now appeared more commonly in the same frame, it mattered more that they were separated by a “black box” that obscured weeks 2–4, between the oldest embryos that could be cultured after IVF and the youngest accessible from terminations.^60^ Research on stem cells has aimed to close this gap and promises more radical change.

## Stem-Cell Models

Tissue culture had long offered models of cell behavior outside bodies (Landecker 2007). Then scientists persuaded hES cells to differentiate into any cell type. Hope and hype about regenerative medicine drove the foundation of institutes, companies, and clinics. Entanglement in the abortion controversy polarized debate (Geesink et al. 2008; Prainsack et al. 2008; Benjamin 2013; Thompson 2013).

Some developmental biologists resented stem-cell science as lacking a distinguishing framework but mushrooming to dwarf their field as it lost status. Others embraced stem cells as a means to embryological knowledge that could also improve ways of deriving the cells (Niakan and Eggan 2013; Zhu and Huangfu 2013). Few expected that ES cells, initially unable to reproduce spatial organization, would “yield information […] of direct relevance to the mechanisms of patterning, axis formation or segmentation” (Pera and Trounson 2004). But as the focus expanded from pluripotency and reprogramming, stem cells were indeed used “to model cell patterning and morphogenetic events in space and time” (National Academies of Sciences, Engineering, and Medicine 2020, p. 8).

Building on mouse work (Martin and Evans 1975), hES cells were induced with growth factors to form “embryoid bodies,” adherent cultures representing all three germ layers but at various stages and disorganized (Itskovitz-Eldor et al. 2000). The key to better models of development was to import skills in 3D culture from fields that had earlier generated growths that resemble organs: “organoids.” Cell culturists had shown that dissociated cells will self-organize into similar patterns as in the original tissue, but that their environments shape how they develop. By the 1970s biologists grew cells in 3D gels of extracellular matrix proteins to produce fragments of mouse mammary gland with the branching structure (Simian and Bissell 2017; Caianiello et al. 2023). Stem-cell scientists revived organoids when, for example, they coaxed adult stem cells from the intestinal crypts to generate “mini-guts” in the basement-membrane preparation Matrigel (Sato et al. 2009; Clevers 2016).

**Fig. 7:**
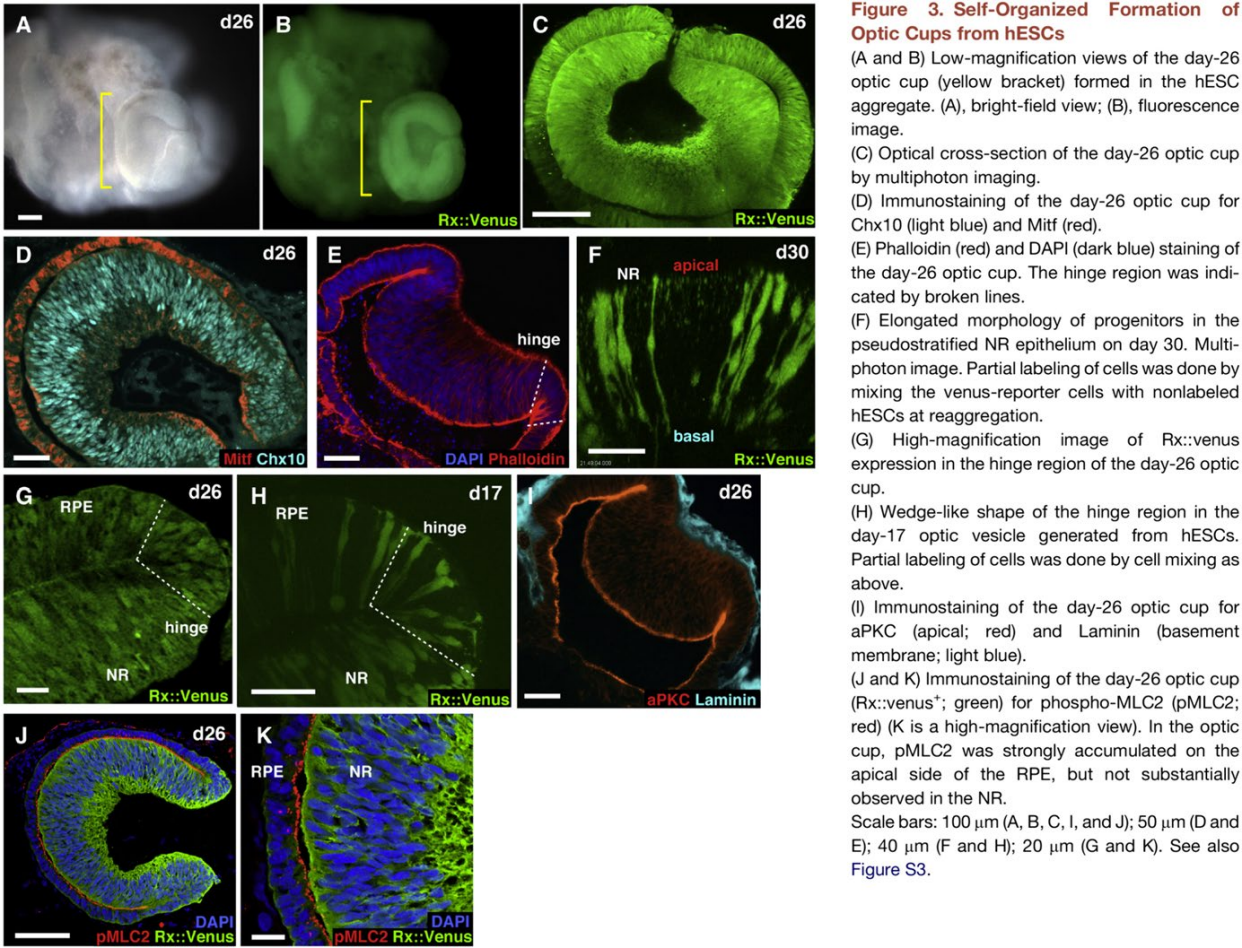
Self-organized formation of optic cups from hES cells, a figure in the influential paper from Yoshiki Sasai’s lab. From Nakano et al. (2012, fig. 3), with permission from Elsevier

The most successful early organoid maker vaunted the power of letting embryonic and induced pluripotent stem cells do what the cells “wanted” in simple culture systems. Yoshiki Sasai drew that lesson from his postdoctoral work, which had reinterpreted the default fate of ectoderm as nerve, not skin, in frogs. At the RIKEN Center for Developmental Biology in Kobe, Sasai’s laboratory cajoled clumps of mouse and then human ES cells to form structures like cerebral cortex and, in 2011–12, optic cups, the human ones larger and with cones as well as rods (Eiraku et al. 2008; Nakano et al. 2012; Costandi 2014; Piccolo 2014; De Robertis 2014) (Fig. 7).

Structures that looked and stained something like eyes and brains sent a mighty message: development must be more canalized and morphogenesis thus easier to produce than expected. At a biotechnology institute in Vienna Madeline Lancaster made cerebral organoids by embedding cells in Matrigel and culturing them in a spinning bioreactor to improve nutrient and oxygen exchange. Containing various brain regions and up to a few millimeters in size, these were strictly too disorganized to count as “miniature human brains,” but the pictures captured imaginations and a model for microcephaly further showed the potential (Brüstle 2013; Lancaster et al. 2013; Lancaster and Knoblich 2014). To answer developmental questions and model diseases, test drugs and toxins, and replace organs, labs have made a “menagerie” of organoids (Vogel 2013). The brain ones, in particular, offer a dynamic approach to human specificity (Benito-Kwiecinski et al. 2021). Researchers hope to alleviate limitations, such as inconsistency, imperfect organization, and off-target effects, by doing more to match the in vivo environment within the self-organization approach. There may be scope to learn from the rival tradition of engineering “organs-on-chips” (Caianiello et al. 2023; Xue et al. 2024).

Embryoids and other “oids” mimicked early embryos and their parts. Biologists and physicists in Brivanlou’s and Eric Siggia’s groups at the Rockefeller collaborated on a quantitative approach. Controlling colony size with spatial constraints, they seeded hES cell suspensions into circular micropatterns, within which the aggregates self-organized into ordered arrays of germ layers that responded to patterning signals with “gastrulation-like events” (Warmflash et al. 2014). Then tissue engineer Nicolas Rivron combined stem cells in microwells to generate “blastoids,” or model blastocysts, in mouse and (alongside other groups) in human (Rivron et al. 2018a; Kagawa et al. 2022; Martinez Arias 2023, pp. 273–279). These can be used to study not just early embryogenesis but also implantation. Trophoblast organoids, which resemble the placenta-forming tissue that develops from the trophectoderm, promise to open up analysis of maternal–fetal interactions (Turco et al. 2018).

**Fig. 8:**
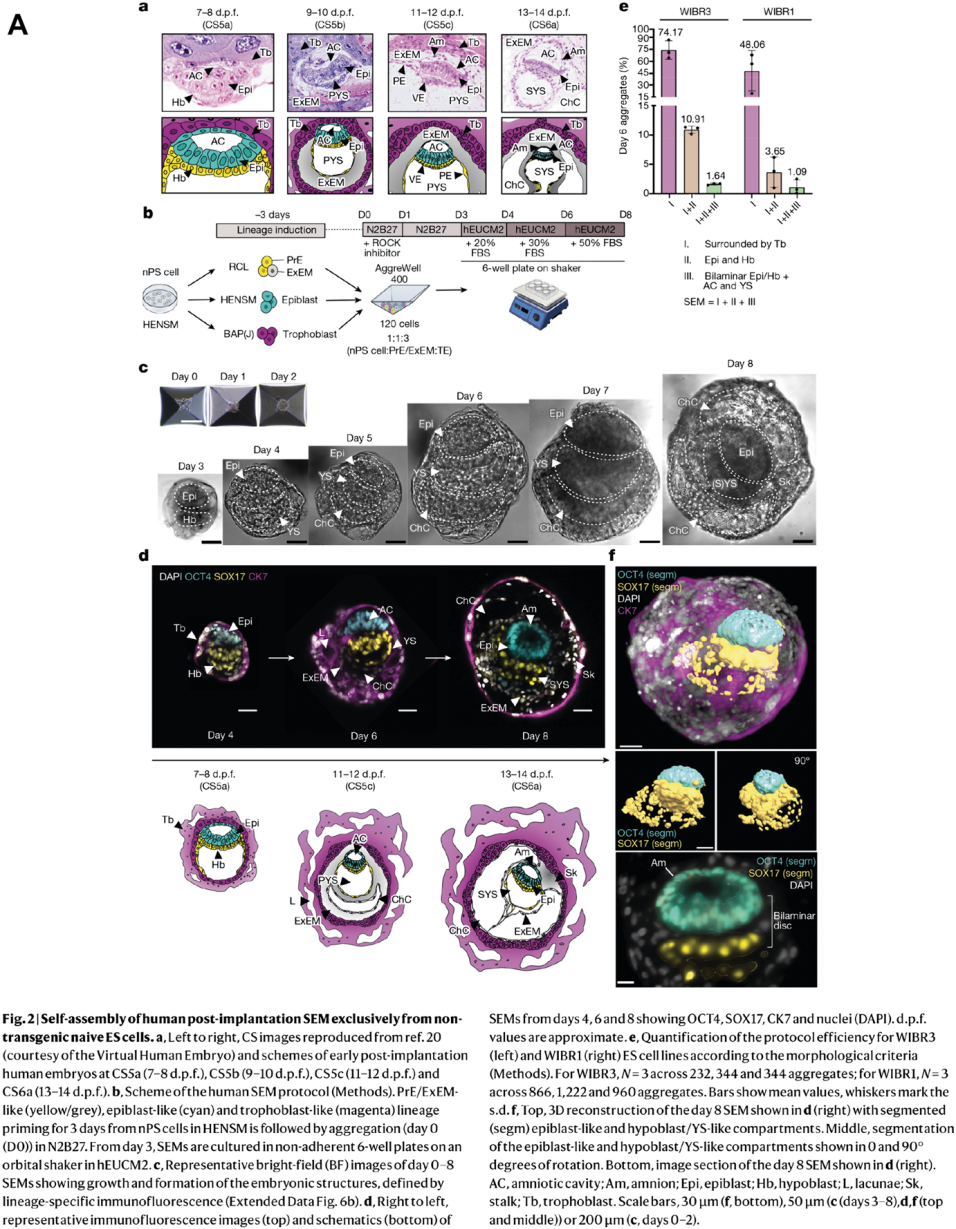

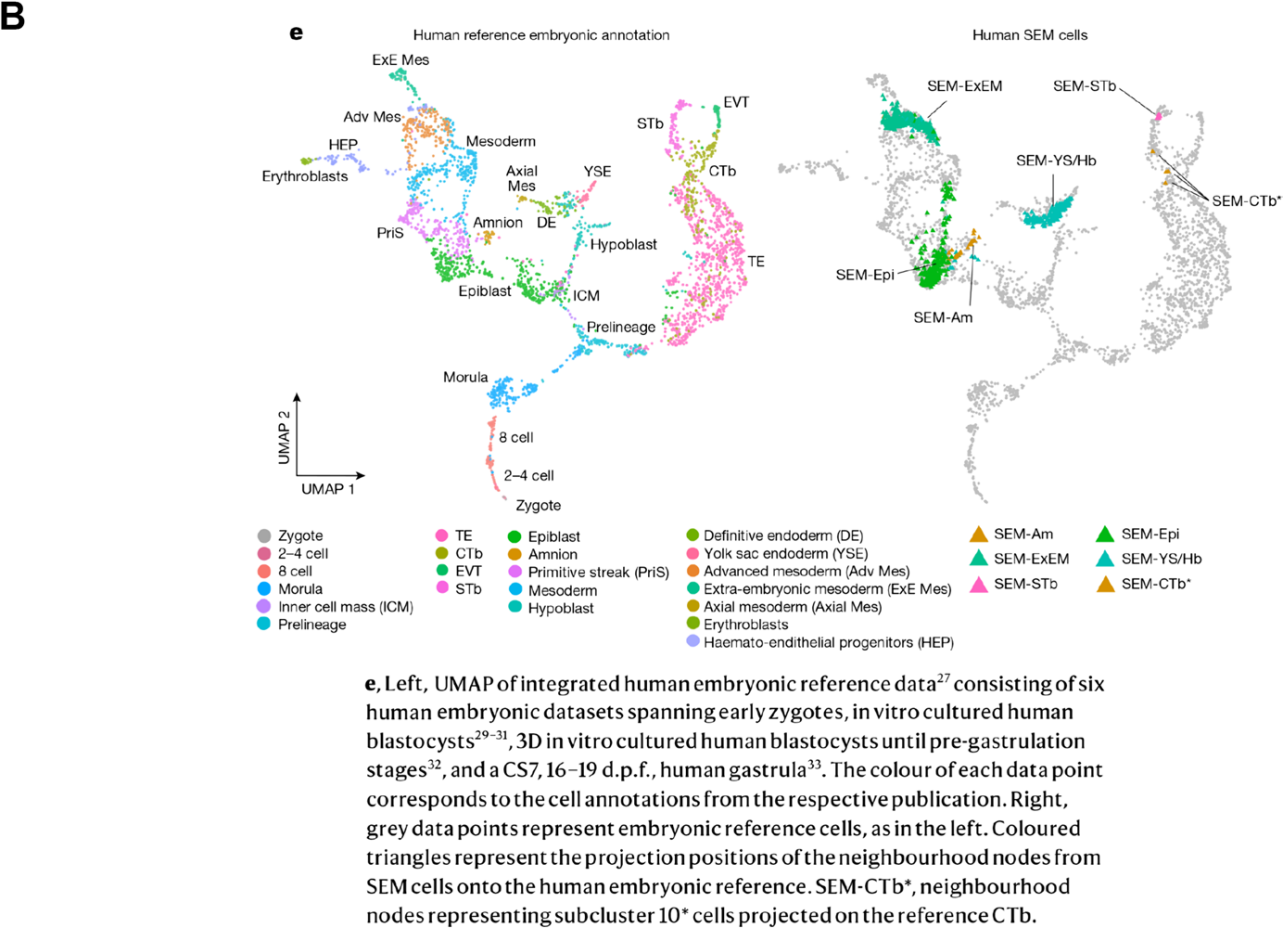
Morphological and molecular characterization of postimplantation embryo models from hES cells in Jacob Hanna’s lab. **A** Comparison with Carnegie stages from the Virtual Human Embryo, scheme of protocol, bright-field microscopy, and immunostaining. **B** Comparison of single-cell transcriptome data, reference (left) and model (right), using UMAP, a dimensionality reduction technique, to show largely cells from the three expected lineages. From Oldak et al. (2023, figs. 2 and 6e) under a Creative Commons license, https://creativecommons.org/licenses/by/4.0/

Such systems face ethical challenges still. For although designed to bypass the barriers to studying humans, the need for regulation, and thus public engagement, became more obvious the more embryo-like the models proved (Rivron et al. 2018b; Foreman et al. 2023). Some labs are nevertheless racing to develop from scratch structures that imitate the most complete and advanced conceptuses they can, most successfully Jacob Hanna’s laboratory at Israel’s Weizmann Institute (Oldak et al. 2023) (Fig. 8).

The ultimate test of normality—replacement in a uterus—is forbidden, so as with the extended culture of IVF embryos, bold claims depend on the unshowy infrastructures that have enabled comparison. Carnegie Collection sections represent morphology. Molecular identification uses lineage markers and transcriptomes lined up with reference data from IVF zygotes and blastocysts and the HDBR gastrula (Fig. 8). The mobilization as standards, in a paper on stem-cell models, of a digitized archival collection and analyses of embryos from fertility clinics and a biobank exemplifies the traditions that are feeding into human developmental biology. Calibration is complicated by normal variation, a hoary issue in embryology that has plagued IVF (Hopwood 2005; Rugg-Gunn et al. 2023), and by wastage rates (Macklon et al. 2002; Jarvis 2017). How to judge in vitro systems when most eggs fertilized in vivo do not make it to birth?

Heroic attempts to prepare whole “synthetic embryos,” as most researchers have learned not to call them, appeal to the ideal that we know what we can make. The main alternative is using culture systems to dissect aspects of development in reproducible ways. Three-dimensional “gastruloids” break symmetry and elongate along the anteroposterior axis (Moris et al. 2020; Martinez Arias 2023, chap. 8). Models of segmentation and somitogenesis similarly give unprecedented spatiotemporal control, with whole dishes of cells corresponding to a small region of an embryo and the capacity to turn gene expression on and off at will (Miao et al. 2023; Yamanaka et al. 2023).

Here, too, the species politics changed. Stem-cell models were made in nonhuman primates and other mammals in which culture could be prolonged. But as researchers followed the funding from mouse to human, many decided that, unless they needed the genetic tools, they could drop the rodents and just induce pluripotency in human fibroblasts. Importantly, mouse naive ES cells are only pluripotent but human ones are totipotent, so blastoids complete with trophoblast and primitive endoderm can be made from them with ease (Guo et al. 2021). Groups that use both species have found human postgastrulation development harder to produce.^61^

Developmental biologists still wanted embryos to provide a standard, or “ground truth” as they called it. Only now some argued for use of the in vitro systems not just to learn the similarities and differences that make us human, or for medical reasons, but to address basic questions in vertebrate developmental biology. Olivier Pourquié, whose Harvard lab studies segmentation in chicks and human organoids, stressed in an interview in 2023 that, thanks to medical investment, humans offer an “incredible database of mutations” and “the most perfect [genome] sequence […]. So in theory the best model organism is human. The problem is that you can’t do experiments, but now you could do experiments.” Human organoids, generated at low cost, were ideal for investigating “fundamental principles that would be hard to study in model organisms.” For Pourquié, clinical relevance is now “the cherry on the cake.” “It’s a great system and it just happened to be human.”^62^

This thought followed a decade when stem-cell-based embryo models helped work on humans gain autonomy and various strands of innovation were woven together.

## From Stem Cells to Human Developmental Biology

Scientists studying human embryos in the disciplines that revived research on them have yet to acquire a group identity as strong as the old model-organism communities. But subcultures did interact. In 1997 the Newcastle geneticists, who went to meetings with HDAC in Washington, edited a book that adapted the Carnegie heritage and included studies of pre- and postimplantation gene expression.^63^ Over the past ten years the rapprochement with stem-cell science has launched a more general human developmental biology.

Between 1980 and 2000, while anatomy and reproductive biomedicine focused on humans as a matter of course, developmental biologists discovered much of the molecular basis of pattern formation and cellular differentiation in model organisms.^64^ These dominate a 1994 issue of *Science* magazine devoted to the present and future of the field; the lone article on the clinic is about growth factors as drugs.^65^ When the tide turned and it was time to deliver for the paying public, some moved into stem-cell science and pioneered cell therapies, doing developmental biology while attempting to cure disease (Melton 2016). But the impact factors of developmental biology journals fell.

Developmental biologists tended to teach that because “all animals use very similar mechanisms […] we really can learn about human development by understanding how it happens in the fruit fly, zebrafish, frog, or mouse” (Slack 2006, p. 3). But model organisms were threatened by calls for research ready for clinical application or “translational” science (Spradling et al. 2006, p. 2032). That term, invented in the early 1990s following the failures of the US War on Cancer, spread in the 2000s (National Institutes of Health 1994). Rallying the troops, the drosophilist chairman of the Gurdon Institute in Cambridge suggested that human organoids could complement the model systems (St. Johnston 2015).

**Fig. 9:**
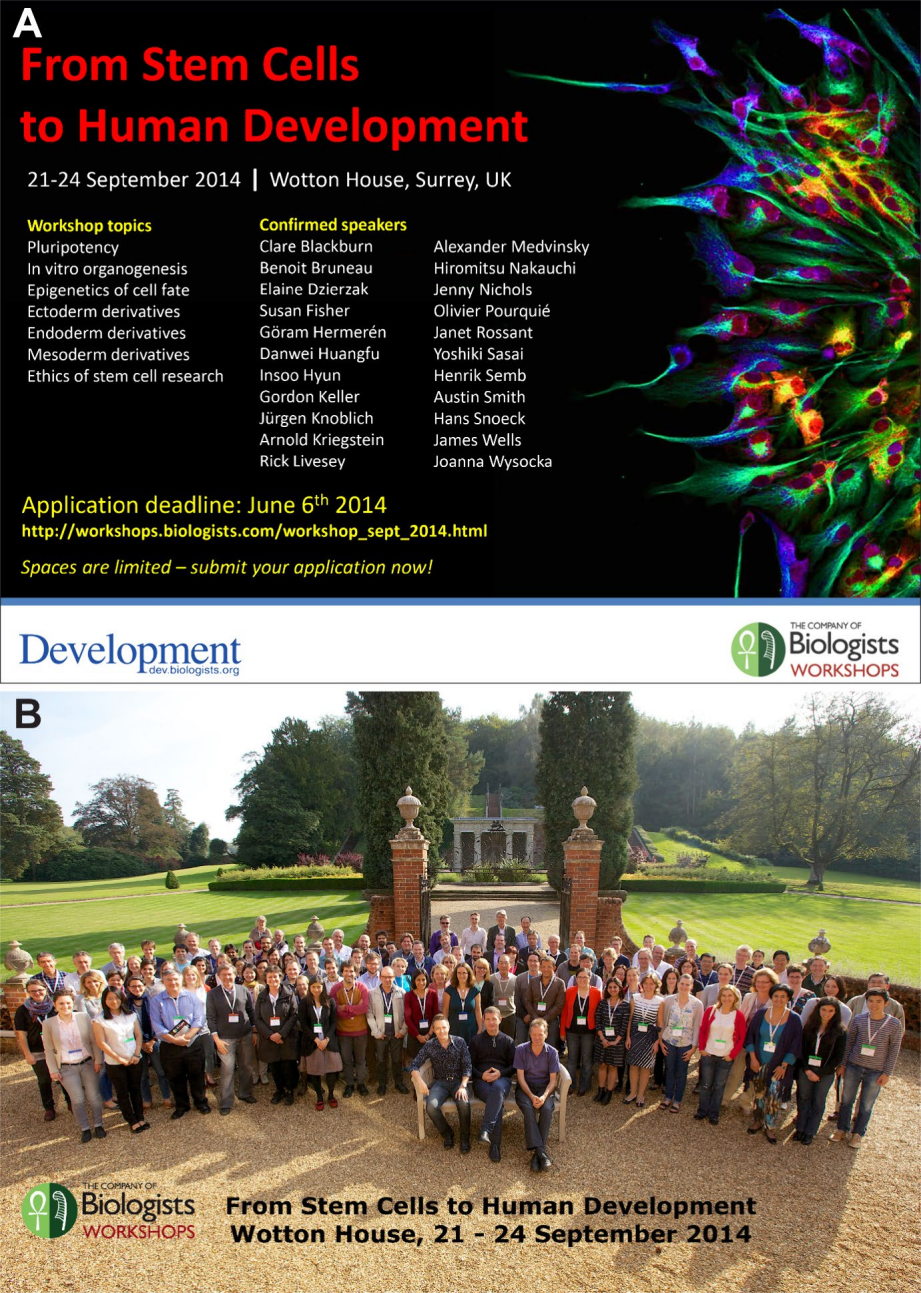
The 2014 Wotton House meeting. **A** Poster listing headline speakers, most of them from the United States, then the United Kingdom, three-quarters men, and including two ethicists (Göran Hermerén and Insoo Hyun). The image, by Troy Ghashghaei and Huixuan Liang, shows a primary neuronal stem-cell culture in which fluorescently labeled proteins mark stem cells (orange/yellow) and their neuronal “offspring” (blue, green, purple). **B** Group photo with the organizers Benoit Bruneau, Olivier Pourquié, and Austin Smith sitting at the front. Katherine Brown, who as executive editor of *Development* was much involved in the planning, stands just behind them in the blue-green dress. Reproduced by permission of the Company of Biologists

In September 2014 Pourquié, as editor-in-chief of *Development*, the leading European journal of developmental biology, led fellow editors in organizing a landmark workshop, funded by its publisher, the Cambridge-based nonprofit the Company of Biologists, at Wotton House in Surrey (Fig. 9). “From stem cells to human development” served his strategy of raising the profile of the journal among stem-cell scientists. Irked by Irving Weissman, who as president of the International Society for Stem Cell Research celebrated their novelty and independence, Pourquié insisted that stem cells belonged in his discipline. It needed them lest it become passé, he argued, while differentiating cells and building organs for regenerative medicine was “an application of developmental biology” (Pourquié 2012). The meeting brought together stem-cell researchers with those working in vivo on humans and mice.^66^ The event became a poignant tribute to Sasai, who was scheduled to speak, when he committed suicide following a fraud case involving a collaborator on an unrelated project (Medvinsky and Livesey 2015).

Pourquié shared “the impression of witnessing at first hand the emergence of this new field of human developmental biology” because stem-cell models would “open the door to […] experimental study” (Pourquié et al. 2015). Skeptics remembered human embryology as old-fashioned and were attached to model organisms. But stem cells offered insight into the mechanisms that developmental biologists sought—and by experiment, their favorite method. The workshop inaugurated a biennial series, which branched out from stem cells, and special issues of *Development*. From 2017 a section of regular issues collected human development papers. A 2023 meeting reversed the emphasis—“From embryos to stem cell models”—and staged encounters between anatomists and stem-cell scientists.^67^ The field is hot because it can mobilize medical, commercial, and intellectual promises. Typically of stem-cell biology, there is more than the usual secrecy and hype but also much-needed discussion of regulation.^68^

The HDBR had already promoted the name human developmental biology. Papers in *Nature*, *Science*, and *Cell*, prizes, and jobs raised its profile. The term fit the disciplinary identity of readers of *Development* and sounded “more modern” than human embryology.^69^ It figured in strategic initiatives involving the HDBR. The Human Developmental Cell Atlas adds the temporal dimension to the Human Cell Atlas, an international enterprise, supported by the MRC in the United Kingdom, that promised a “‘Google map’ of the human body” using high-throughput sequencing of mRNA and chromatin accessibility (Behjati et al. 2018; Haniffa et al. 2021, p. 197). This “multiomic profiling” strives to become an in vivo “benchmark” of normal human development to complement comparative and in vitro approaches, with the tantalizing possibility of computational methods filling the gaps (Haniffa et al. 2023).

Intersecting with the atlas, in 2019 Wellcome gave a consortium of research groups £10M to trace cell lineages and so create “a treasure trove of data and technologies.”^70^ This Human Developmental Biology Initiative consolidated the reasoning of the previous two decades: little is known about human development, which must be studied because there are important differences from mice; the United Kingdom has an ethical supply of conceptuses from fertility clinics and through the HDBR; and omics and organoids promise revolutionary insights that may improve fertility treatments, prevent birth defects, and develop regenerative medicine. Bringing together pre- and postimplantation work and stem-cell models, the focus is on early stages plus cardiopulmonary, neural, and blood and immune systems. Public engagement discusses “what makes us human?” as it fosters dialogue about the research.^71^

Developmental biology textbooks traditionally left our species to anatomical texts for medical students. It was common to hold out an understanding of “how we humans develop” as “one of the ultimate aims of the science,” but to say almost nothing more about us (Wolpert et al. 2011, pp. 1–2). In the last few years major texts met the interests of students, colleagues, and publishers by expanding coverage a little or by adding chapters on early human development. They now list humans among the model organisms included, aware that this is stretching the term.^72^

Pressed in specific fields in the 1980s and 1990s, the argument for studying human embryos directly succeeded generally in the early 21st century. But even scientists who rode this wave have lamented a lack of money for mice—and well they might.^73^ Researching human development became an active field of biomedicine because of not despite its dependence on models.

## Conclusion

It might seem obvious today that research has to tackle humans to correct or confirm conclusions from other species and reap the medical rewards; that only human embryos can reveal what makes us human; and even that fundamental features of vertebrate development should be analysed in embryo-mimicking assemblages of human cells. A historical perspective restores a sense of surprise about the success of these claims and uncovers their long-term significance. This article has explained the growing attractiveness of studying humans directly, showing the facilitating effects of sources of material, calls for clinical relevance, and methods of analysis. It has dissected arguments for this choice and reconstructed the roles that models have played.

Shifts in supply and demand drove the revival of research on human embryos. The programs that are coalescing used archival collections and fresh material to meet the desire for medical knowledge and (pre)translational science. Justifications customarily appealed, with Keibel, to “the fact that we are human beings” as well as “importance to physicians.” But in the 1980s the UK campaign for work on living preimplantation embryos pressed the utilitarian case to avoid any hint that mere curiosity might drive controversial experimentation. From the mid-1990s biologists reasserted self-knowledge as a goal, while recognizing that funders want translation (compare McLaren 1986 and Rossant 2024). The atmosphere of well-resourced excitement is tempered but also heightened by ethical concern.

Where the Carnegie embryologists modeled developmental anatomy, their successors are working through the opportunities opened up by culture and reprogramming, imaging and omics. Slotting human embryos into data-centric science made the most of scarce material and has shared features of postgenomic, digital biomedicine at the turn of the millennium. Knowledge of health, risk, and identity is distributed in a networked world and promises therapeutic intervention through bespoke cells and organs as well as molecules. Practitioners have engaged publics and politicians over the regulation of collecting and culture.^74^

Genomics mired itself in broken promises about genes and disease, but omics is aiding human developmental biology to characterize and so manipulate cells (Martinez Arias 2023). Studying them depends as much on advanced imaging, and culture systems designed for it, as on sequencing nucleic acids. But most research in human developmental biology has highlighted genes, gametes, and embryos. More might join sciences of reproduction in embracing environmental effects (Lappé et al. 2019; Van Wichelen and Keaney 2022). Systems have been invented to investigate implantation—long recognized as a limiting step in IVF—as well as placentation and exposures during development. With individual embryos still as crucial as arrays of embryoids, standards-setting has to address the old challenges of normal variation and natural wastage.

Models led the way to work on humans. The digitization of existing collections proceeded independently, but most initiatives depended on models while struggling against entrenched interests in them and then stealing the show. Model organisms initiated scientists into what could be known, and the skills and infrastructures needed to discover it. Mice became a more important foil than were monkeys at the Carnegie Department or rabbits in reproductive biology. The imperfection of the mouse model enabled the continual discovery of new aspects of adequacy and insufficiency. In a constant back and forth the opportunities for emulation increased as research on the model forged ahead. Where mice lose out as too unlike humans, advocates have positioned other surrogates, their funding still minuscule, to move up should the ground continue to shift. Though unlikely to take over, these could enrich comparisons among mammals to triangulate human specificity.^75^

Stem-cell models are modifying these politics of species choice. Unambiguously human, they allow experimentation on simulacra of developing tissues and organs subject to negotiable limits. The history of the mouse–human duo suggests that stem-cell models could be most productive (and acceptable) not when they approximate embryogenesis as a whole, but when they resemble human embryos only in certain respects. Be that as it may, they are flourishing because of the constraints on human research. They promise to loosen its dependence on other animals and to strengthen the senses in which the species to be modeled is, paradoxically, becoming a model. How far, with the necessary public consultation and consent, will investment and ingenuity take it? Whatever the answer, research directly on human development will surely continue to rely on models—organisms as well as in vitro systems—that have themselves been, and are being, transformed.

## Footnotes

1 Compare anatomists O’Rahilly (1988) and Gasser et al. (2014) with biologists Van Blerkom (1994), Niakan et al. (2012), and Brivanlou and Gleicher (2021); but investigators of preimplantation embryos acknowledge the legacy of the Carnegie Department (on which, see Maienschein et al. 2004 and Morgan 2009) more than they did.

2 The product of human fertilization is a conceptus, which gives rise to the embryo proper and the placenta. Though the term “conceptus” should, strictly, be used for preimplantation stages (called “pre-embryos” by research advocates in the 1980s), because these always include cells that will form the placenta, I shall also follow standard usage and refer to embryos up to eight weeks postfertilization, and then to fetuses. Ages are from fertilization unless otherwise stated, two weeks younger than the gestation weeks that obstetricians time from the start of the last menstrual period.

3 I focus on embryo research, which may extend into the fetal period, and not on teratology, placentology, or fetology, let alone neonatology or pediatrics.

4 On research opportunities created by medical infrastructure, see Clarke (1987), Oudshoorn (1994), and Bangham (2020); on postgenomics, Richardson and Stevens (2015), Reardon (2017), and Van Wichelen and Keaney (2022); and, on being human, Rees and Sleigh (2020).

5 For model organisms as reference points for investigating other species, see Leonelli (2016, chap. 6) and, for the ongoing revalidation of models, Nelson (2018).

6 This follows GenomeWeb News, “Sydney Brenner urges cancer researchers to consider ‘bedside to bench’ approach,” GenomeWeb, April 15, 2008, https://www.genomeweb.com/archive/sydney-brenner-urges-cancer-researchers-consider-bedside-bench-approach; see also Brosnan and Postma (2017).

7 On earlier disciplinary identities in embryo research, see Hopwood (2009, 2019).

8 Quotations from Hertig to Streeter, February 26, 1941 (saying that it was not then “working full-blast”), in Arthur Hertig Papers (H MS c11), Center for the History of Medicine, Countway Library, Harvard University, box 5, folder 40.

9 Anon. (photos by Chester F. Reather), “How Life Begins,” *Science Illustrated* 1 (July 1946): 15–20.

10 For the deal, which collapsed when anatomist Ernest Gardner, who had brokered it as dean of the medical school, moved to Davis, see Gardner to University President William Keast, February 24, 1969 (quotations); Keast to James Ebert, May 1, 1969; and Ebert to Keast, May 7 and 12, 1969, in University Vice President for Academic Administration Winfred Harbison Records, Walter P. Reuther Library, Archives of Labor and Urban Affairs, Wayne State University, box 71, folders 6–7.

11 Klaus Hinrichsen to Erich Blechschmidt, July 11, 1988, in ring binder, Institut für Anatomie und Embryologie, Zentrum Anatomie, Universität Göttingen.

12 Editorial, “Embryos outside the body,” *Nature* 223 (1969): 1041–1044, https://doi.org/10.1038/2231041a0.

13 Editorial, “The future of the test-tube baby,” *Nature* 299 (1982): 475–476, https://doi.org/10.1038/299475a0.

14 *Report of the Committee of Inquiry into Human Fertilisation and Embryology* (London: Department of Health & Social Security, 1984), p. 62.

15 D. G. Whittingham, “Personal comments on Warnock Committee report,” [1984], p. 4, in Progress: Campaign for Research into Human Reproduction archive, LSE Library (hereafter **LSEP**), box 1, folder 1. On the act, see Kirk and Myelnikov (2022).

16 *Report of the Committee of Inquiry*, pp. 91–92.

17 Progress, *Why pre-embryo research is needed*, [1985?], LSEP, box 2, folder 6; see also Progress, *Freedom to choose: Research into infertility and congenital handicap*, [1985], LSEP, box 2, folder 6; and MRC, *Why pre-embryo research? What you need to know*, [1986?], LSEP, box 2, folder 5.

18 Similar work at the Animal Research Station in Cambridge had shown activation at four cells in pigs and eight in sheep; mammalian embryologists interacted across species and in this city intensely.

19 “Of mice and men,” *MRC News*, no. 38 (March 1988): 10–11; see also Vines (1989) and MRC, *Human fertilisation and embryology*, [1990], LSEP, box 13, folder 46.

20 *Human Fertilisation and Embryology Act 1990* (London: HMSO, 1990), pp. 35–36; see also *Human Fertilisation and *Embryology* Act 2008* ([London]: HMSO, 2008), p. 60.

21 *Armed Forces* *Institute of Pathology Annual Report 1991*, pp. 228–229; Noe (2004, p. 49). For O’Rahilly’s view, see Don Hilbelink to Delbert Dayton (NICHD), December 10, 1991, in Carnegie Collection of Embryology, Carnegie Records Series, Human Developmental Anatomy Center, National Museum of Health and Medicine (hereafter **HDACCa**), box Ca 146, folder “NIH/Dayton.” In 2011 HDAC relocated to US Army Forest Glen Annex, Silver Spring, MD.

22 Noe to Stocker, “Preliminary proposal, Human Developmental Anatomy Center, AFIP,” November 23, 1990, HDACCa, box Ca 142, folder 6.

23 Gasser to Noe, September 13, 1991, HDACCa, box Ca 145A, folder “Gasser;” Hilbelink to Dayton, December 10, 1991.

24 This is part of the rationale in Gasser and Noe (PIs), “Human Embryo Sections on DVDs for Education,” NIH grant application, revision of R01 HD 37811-01, June 1999, HDACCa, box Ca 147, folder “Gasser grant 1999;” see also Gasser et al. (2014).

25 *Armed Forces* *Institute of Pathology Annual Report 1993*, p. 227.

26 Gasser (1975). In a contrast to liberal reproductive biologists, Gasser shared Blechschmidt’s Catholic antiabortionism: “Curriculum vitae: Raymond Frank Gasser,” January 14, 1991, HDACCa, box Ca 145A, folder “Gasser;” Gasser (1982).

27 Tsiaras (2002); “The Oprah Winfrey Show,” March 21, 2003; Morgan (2009, pp. 217–221).

28 George Michaels, “Technical proposal: Human Embryology Digital Library and Collaboratory Support Tools,” June 30, 1999, HDACCa, box Ca 145A, folder “NIH grant 1993;” J. Mark Pullen, “Final report, Contract N01-LM-0-3508, Human Embryology Digital Library and Collaboratory Support Tools,” June 15, 2003, https://netlab.gmu.edu/visembryo/VEFinalReport.pdf.

29 Gasser (PI), “Human Embryo Sections on DVDs for Education,” NIH grant application, competing continuation of R01 HD 37811-01, June 2001, HDACCa, box Ca 147, folder “Gasser grant 2001.”

30 Gasser et al. (2014); Endowment for Human Development, “The Virtual Human Embryo,” https://www.ehd.org/virtual-human-embryo/, accessed December 13, 2023.

31 Hill (2019); Mark Hill, Human Embryology website, http://tiny.cc/Embryo; Digital Embryology Consortium, https://human-embryology.org, both accessed April 18, 2024; on organism databases, see Leonelli and Ankeny (2012), Leonelli (2016), and Bangham (2019).

32 On biobanking, see Gere (2003) and Fannin and Kent (2015); for “ethical efficiency” with respect to preimplantation embryos, see Hurlbut (2015).

33 Sheldon et al. (2022, pp. 51–55, 135). On supply to stem-cell science, see Pfeffer and Kent (2007), Pfeffer (2008), and Cooper and Waldby (2014, chap. 5); and, on fetopathology, Löwy (2018).

34 A grant application reviews the earlier history: “HDBR_2002-07_WT,” Q11, §2.3, in Andrew Copp’s HDBR papers (hereafter **ACP**). I shall reference other extracts from HDBR grant applications by the filenames of the pdfs Copp shared with me.

35 Totals: Susan Lindsay, short CV, in Lindsay’s personal papers; Copp to author, March 23, 2024. The HDBR has managed the MRC Fetal Tissue Bank collection since 2005: “HDBR_2008-13_MRC,” [extract], §2.3, ACP.

36 “National developmental biology resource,” *Wellcome News*, issue 20, Q3 (1999): 3.

37 Zoom interviews with Andrew Copp, November 22, 2022, and Susan Lindsay, April 2, 2021. The name had figured, differently, in a short-lived US Public Health Service-funded journal: “*Abstracts of Human Developmental Biology*,” *Nature* 190 (1961): 133, https://doi.org/10.1038/190133b0; but “developmental biology” at the NICHD focused on model systems.

38 “HDBR_2002-07_WT,” Q11, §§3.9, 5.3; Lindsay and Copp (2005); Gerrelli et al. (2009, 2015).

39 Lindsay and Copp interviews. Copp made the point strongly: “I have a lot to do with […] neurosurgeons […] and […] they constantly refer to me as an embryologist and I constantly tell them I’m not […] I’m a developmental biologist and I will not be called an embryologist […] because an embryologist is somebody who sits and pipettes preimplantation embryos as a technician effectively.” Compare Slack (1999, pp. 19–20) and Trinkaus (2003).

40 “HDBR_2002-07_WT,” Q11, §3.1; “HDBR_2018-23_MRC,” Case for support, §§1.2 (quotation), 2.1, ACP.

41 Copp interview.

42 “HDBR_2023-27_MRC,” Case for support, §§1.2, 2A, ACP.

43 “HDBR_2002-07_WT,” Q11, §5.2b.

44 “HDBR_2002-07_WT,” Q11, §5.5; “HDBR_2008-13_MRC,” §2.8.

45 “HDBR_2002-07_WT,” Q11, §5.4; “HDBR_2023-27_MRC,” Case for support, §2B.

46 “HDBR_2023-27_MRC,” Case for support, figs. 1–3.

47 “HDBR_2018-23_MRC,” Case for support, §1.5.

48 Boonstra (2016); MacDuffie et al. (2021); “Laboratory of Developmental Biology,” project number 2R24HD000836-57, NIH RePORTER, https://reporter.nih.gov/project-details/10732157, accessed April 26, 2024; “The Dutch Fetal Biobank,” Amsterdam University Medical Centers, https://www.3dhumandevelopment.com/dutch-fetal-biobank/, accessed September 24, 2023; “Biobank,” HuDeCA, https://hudeca.com/?page_id=1632, accessed March 9, 2024.

49 At the same time genomics lowered barriers to work on a wide range of long-ignored organisms. While NIH poured money into the dominant models, the US National Science Foundation funded programs that critiqued over-reliance on species selected for laboratory life and tackled evolutionary and ecological questions they were poorly suited to address; see Gilbert (2009).

50 Burn and Strachan (1995); Strachan et al. (1997a, 1997b). For early studies of gene expression in human embryos, see Gatherer et al. (1990) and Hertle et al. (1991).

51 Quoted in Burn and Strachan (1995, p. 4); the application was probably for Strachan et al.’s first Wellcome grant, “Studies of gene expression in early stage human embryos,” October 1996–September 1999. Human geneticists could struggle to persuade colleagues of results different from those in mice; see Migeon (2003, 2016, chap. 7) and Lee (2003).

52 Lindsay interview.

53 Lindsay interview.

54 Sarma et al. (2005); Kerwin et al. (2010); “HDBR_2013-18_WT,” Q10a, §iii.

55 Alain Chédotal, “3D analysis of human embryonic development,” seminar, University of Cambridge, June 17, 2022; visit to Chédotal lab, June 23, 2022; Inoue et al. (2022).

56 Tyser et al. (2021). The HDBR gene expression service now offers spatial transcriptomics, which attaches a cryosection to a slide seeded with bar-coded primers for reverse transcription and so can map the mRNA data back to positions in the section; see “HDBR_2023-27_MRC,” Case for support, §2C.

57 UK donations collapsed over the past 15 years: Hannah Devlin, “Call to help UK IVF patients donate unused embryos after shortage hinders research,” *The Guardian*, December 6, 2023, https://www.theguardian.com/science/2023/dec/06/call-to-help-ivf-patients-donate-unused-embryos-after-shortage-hinders-research. On a US biobank, see Hurlbut (2015) and Cromer (2018).

58 “Humanized” mice (Davies 2012) became crucial to preclinical testing, including of cell therapies, but have played little role in the basic research surveyed here.

59 Niakan, conversation in Cambridge, December 12, 2023.

60 Rossant (2016); Hopwood (2022, pp. 24–25). The new term had been used of early pregnancy loss; see Macklon et al. (2002). It was preceded by, for example, “Dark Ages” (McLaren 1989, p. 85) and “inaccessible window” (Burn and Strachan 1995).

61 Alfonso Martinez Arias, conversation over Zoom, February 2, 2024.

62 Interview with Olivier Pourquié, Hale Building for Transformative Medicine, Brigham and Women’s Hospital, Boston, October 24, 2023.

63 Strachan et al. (1997b); for the meetings, Lindsay interview.

64 Anatomists also took comparative approaches and selected the right species for a job.

65 Editorial and a total of 15 news and perspectives pieces and articles, “Frontiers in Science: Development,” *Science* 266 (1994): 523, 561–614.

66 For announcement, programme, and photos, see Company of Biologists, “From stem cells to human development,” https://www.biologists.com/meetings/from-stem-cells-to-human-development-2014/, accessed February 3, 2024.

67 For example, Christoph Viebahn and Jacob Hanna; Cold Spring Harbor Asia, “Human development: From embryos to stem cell models, Awaji, Japan,” March 5–10, 2023, https://www.csh-asia.org/?content/505, accessed February 3, 2024.

68 Participants at the Awaji meeting complained about secrecy; see also Huch et al. (2017), Ball (2023), and Rugg-Gunn et al. (2023, p. 4).

69 Quotation from Pourquié interview.

70 Wellcome, “Wellcome-funded initiative to unlock secrets of human development,” July 25, 2019, https://web.archive.org/web/20220705052444/https://wellcome.org/press-release/wellcome-funded-initiative-unlock-secrets-human-development, accessed April 26, 2024.

71 Gurdon Institute, “Human Developmental Biology Initiative,” https://www.gurdon.cam.ac.uk/our-research/hdbi/, accessed September 13, 2022.

72 Wolpert et al. (2019, pp. 131–138); Slack and Dale (2022, chap. 12); Barresi and Gilbert (2024, chap. 14). Jonathan Slack and Leslie Dale took in an organism “very difficult to study directly” because “it has become increasingly clear that there are […] many differences from the mouse which may be important for various practical purposes” (p. 203). Scott Gilbert, whose text, first published in 1985, had long referred to humans, “avoided writing this chapter for over two decades.” Although trained in pediatric genetics, he “felt,” as an advocate of evolutionary and ecological developmental biology, “that the book was already too vertebrate-centric.” He gave in to his “co-author, the students, the professors, and the publisher” because “there were finally data that I could write about” (referee’s report on this article, March 20; Gilbert to author, April 25, 2024). Compare the most mechanistic human embryology text: Schoenwolf et al. (2021).

73 Zernicka-Goetz and Highfield (2020, p. 225); Emma Rawlins in “How organoids help us understand ourselves and treat diseases,” Cambridge Festival, March 29, 2021, https://www.youtube.com/watch?v=mqe9Tcst2R0, at 45:09–59, also for the point that mice could provide in vivo models for testing the capacities of human cells.

74 Some shifts match those outlined by Clarke et al. (2003).

75 For the difficulty of establishing a new system, see Canizo et al. (2024).
